# Plasma ctDNA is a tumor tissue surrogate and enables clinical-genomic stratification of metastatic bladder cancer

**DOI:** 10.1038/s41467-020-20493-6

**Published:** 2021-01-08

**Authors:** Gillian Vandekerkhove, Jean-Michel Lavoie, Matti Annala, Andrew J. Murtha, Nora Sundahl, Simon Walz, Takeshi Sano, Sinja Taavitsainen, Elie Ritch, Ladan Fazli, Antonio Hurtado-Coll, Gang Wang, Matti Nykter, Peter C. Black, Tilman Todenhöfer, Piet Ost, Ewan A. Gibb, Kim N. Chi, Bernhard J. Eigl, Alexander W. Wyatt

**Affiliations:** 1 Department of Urologic Sciences, Vancouver Prostate Centre, University of British Columbia, Vancouver, BC Canada; 2 Department of Medical Oncology, BC Cancer, Vancouver, BC Canada; 3 Faculty of Medicine and Health Technology, Tampere University and Tays Cancer Centre, Tampere, Finland; 4 Department of Radiation Oncology and Experimental Cancer Research, Ghent University Hospital, Ghent, Belgium; 5 Department of Urology, University Hospital Tübingen, Tübingen, Germany; 6 Department of Pathology and Laboratory Medicine, BC Cancer, Vancouver, BC Canada; 7 Studienpraxis Urologie, Nuertingen, Germany; 8 Medical School, Eberhard-Karls-University Tübingen, Tübingen, Germany; 9 Decipher Biosciences, Inc., Vancouver, BC Canada

**Keywords:** Cancer genomics, Bladder cancer

## Abstract

Molecular stratification can improve the management of advanced cancers, but requires relevant tumor samples. Metastatic urothelial carcinoma (mUC) is poised to benefit given a recent expansion of treatment options and its high genomic heterogeneity. We profile minimally-invasive plasma circulating tumor DNA (ctDNA) samples from 104 mUC patients, and compare to same-patient tumor tissue obtained during invasive surgery. Patient ctDNA abundance is independently prognostic for overall survival in patients initiating first-line systemic therapy. Importantly, ctDNA analysis reproduces the somatic driver genome as described from tissue-based cohorts. Furthermore, mutation concordance between ctDNA and matched tumor tissue is 83.4%, enabling benchmarking of proposed clinical biomarkers. While 90% of mutations are identified across serial ctDNA samples, concordance for serial tumor tissue is significantly lower. Overall, our exploratory analysis demonstrates that genomic profiling of ctDNA in mUC is reliable and practical, and mitigates against disease undersampling inherent to studying archival primary tumor foci. We urge the incorporation of cell-free DNA profiling into molecularly-guided clinical trials for mUC.

## Abstract (web summary)

In metastatic urothelial carcinoma, it has not been established whether circulating tumor DNA (ctDNA) can replace archival primary tissue to assess mutations and biomarkers. Here, the authors show high mutation concordance between ctDNA and tumour tissue, with high consistency in serial samples.

## Introduction

Bladder cancer is the tenth most common cancer and the sixth in men, where incidence and mortality are higher^1^. At diagnosis, 25% of patients present with aggressive muscle-invasive or metastatic urothelial carcinoma (mUC), the latter being invariably lethal^2^. Fortunately, there are now several approved therapies in the metastatic setting, including platinum chemotherapy, immune checkpoint inhibitors, anti-fibroblast growth factor receptor (FGFR)-targeted therapy, and an antibody–drug conjugate targeting Nectin-4^3–5^. However, response rates are highly variable and treatment side effects can be significant, so practical biomarkers to predict patient benefit are urgently required.

Primary muscle-invasive bladder tumors have the third highest mutation rate of all studied cancers^6^. Inter-patient heterogeneity is high, but recurrent gene alterations include mutation to chromatin modifiers, cell-cycle regulators, members of the phosphoinositide 3-kinase pathway, and the *TERT* promoter^7^. As such, genome and transcriptome characterization offer opportunities for patient stratification; tumor mutational burden (TMB), *FGFR3* activation, *ERCC2* mutation, PD-L1 expression, and RNA subtyping have shown promise for clinically relevant segmentation of primary bladder cancer^4^. However, due to the limited availability of metastatic tissue, it remains unclear whether signatures derived from primary tumor tissue are representative of disseminated disease.

In other cancers, somatic alterations detected in plasma circulating tumor DNA (ctDNA) are associated with therapy resistance and response^8,9^. We and others have previously shown, in small patient cohorts, that ctDNA can be detected and characterized in mUC^10–13^, and may have clinical value^14,15^. Given the heterogeneity of primary bladder cancer, and the evolutionary pressures of intervening therapy, it is plausible that a ctDNA-based profile better reflects contemporary late-stage mUC than archival primary tissue. However, tissue remains the gold standard and no studies have comprehensively evaluated differences between tumor tissue and ctDNA.

Here, we examine ctDNA and archival tumor tissue from a large cohort of patients with mUC. We show that ctDNA abundance is highly prognostic for patient outcomes and that somatic profiles derived from ctDNA are supported by those from patient-matched tissue. Our results encourage efforts to integrate liquid biopsy technology into the management of patients with mUC.

## Results

### High ctDNA levels independently predict aggressive disease

**Fig. 1:**
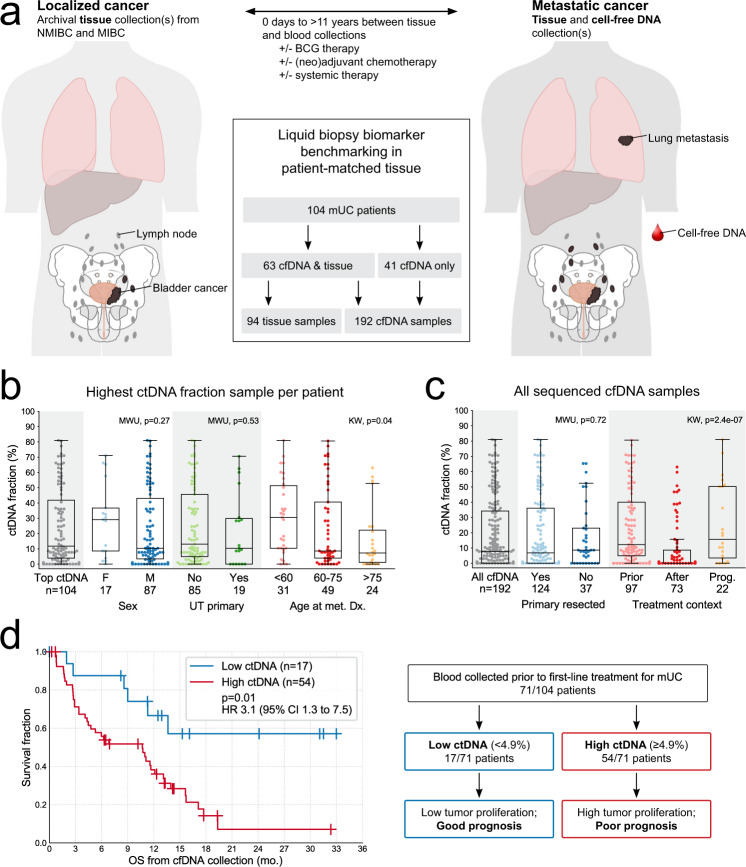
Cohort summary and abundance of circulating tumor DNA (ctDNA). **a** Cell-free DNA (cfDNA) and tissue samples were collected from 104 metastatic urothelial carcinoma (mUC) patients. Anatomy diagram obtained from Cancer Research UK/Wikimedia Commons, available under a Creative Commons Attribution-Share Alike 4.0 International license: https://commons.wikimedia.org/wiki/File:Diagram_showing_advanced_bladder_cancer_CRUK_441.svg. **b** Abundance of ctDNA in relation to patient characteristics. Only the highest ctDNA fraction sample from each patient is shown. **c** Impact of treatment status (at the time of cfDNA collection) on ctDNA abundance. Prior cfDNA collected pre-treatment initiation, after cfDNA collected post-treatment initiation, and Prog. cfDNA collected near the time of documented disease progression (see “Methods” section). **d** Kaplan–Meier survival analysis in 71 mUC patients with cfDNA collected prior to first-line systemic therapy. The highest ctDNA fraction sample from each patient is represented if multiple pre-treatment samples were available. Stratification is based on the 25th percentile across the represented samples (4.9%). Statistical significance was measured using Cox proportional hazards regression analysis. All boxplots in (**b**) and (**c**) are centered at the median, with the box spanning the first to third quartile, and minima and maxima extending to 1.5× IQR. MWU two-sided Mann–Whitney *U* test, KW Kruskal–Wallis test, and UT upper tract. Source data for (**b**) and (**c**) are provided as a Source Data file.

We collected 192 blood samples from 104 patients during their treatment for mUC (Table 1, Fig. 1a, Supplementary Fig. 1a, and Supplementary Data 1). Most patients provided samples prior to first-line platinum-based chemotherapy or immune checkpoint inhibition (Supplementary Fig. 1b). Plasma cell-free DNA (cfDNA) was subjected to targeted sequencing using a custom 50 or 60 gene panel, to a median unique read depth of 1040×, alongside patient-matched germline (leukocyte) DNA (Supplementary Data 1). Utilizing somatic mutations detected exclusively in the cfDNA, we calculated the proportion of cfDNA that was tumor-derived (ctDNA). In 85% of patients (88/104), the ctDNA fraction was >1% in at least one sample, and in 80 patients protein-altering somatic mutations were identified enabling genomic characterization of the tumor from the blood (Fig. 1b and Supplementary Data 2). The median ctDNA fraction across all 192 samples was 8%, was above 1% in 132 samples (69%; 20% median in these samples), and ctDNA fraction was correlated between temporal patient-matched samples (*n* = 31 pairs, Pearson’s *r* = 0.72, *p* = 5.8e − 06; Fig. 1c and Supplementary Fig. 2a, b). Whole-exome sequencing (WES; median depth 213×) was performed on 49 samples, and independently derived tumor purity estimates from exome-wide copy number profiling were consistent with mutation-based estimates from targeted sequencing (*n* = 30 sample pairs, Pearson’s *r* = 0.85, *p* = 2.5e − 09; Supplementary Fig. 2c, d).

**Table 1:** Clinical characteristics for the metastatic urothelial carcinoma (mUC) patient cohort.

	mUC cohort (*n* = 104)	Subset with tissue retrieved (*n* = 63)	Subset without tissue retrieved (*n* = 41)	Fisher’s exact *p*^a^
Median age at metastatic diagnosis (range)	67 (37–88)	67 (37–88)	67 (44–83)	
Male (%)	83.7	85.7	80.5	0.59
Smoker (%)	70.7	71.7	68.8	0.81
Upper tract involvement (%)	18.3	15.9	22.0	0.45
Metastatic at diagnosis (%)	18.3	12.7	26.8	0.08
Initial histology of invasive component (%)				
Pure urothelial	50.0	42.9	61.0	0.11
Mixed urothelial with variant	10.6	14.3	4.9	0.19
Pure variant	26.9	27.0	26.8	1.00
Unknown	12.5	15.9	7.3	0.24
Curative-intent treatment (%)				
Cystectomy	79.3	72.2	90.9	0.05
Trimodal therapy	9.1	13.0	0.0	0.17
Chemotherapy	43.4	41.2	48.0	0.63
Clinical factors at metastatic diagnosis (%)				
ECOG PS ≥ 2	21.1	20.0	22.5	0.80
Visceral metastases	71.2	66.7	78	0.27
First-line treatment for metastatic disease (%)				
None	13.9	19.4	5.1	0.07
Cisplatin-based	35.6	24.2	53.8	0.0031
Carboplatin-based	17.8	19.4	15.4	0.79
PD-1/PD-L1	22.8	27.4	15.4	0.22
PD-1 + CTLA-4	4.0	4.8	2.6	1.00
Taxane	2.0	1.6	2.6	1.00
Other	4.0	3.2	5.1	0.64
Median days from metastatic diagnosis				
To initiation of first-line treatment^b^	39	43	39	
To first cell-free DNA collection	62	75	41	
Median months of follow-up (range)				
From metastatic diagnosis	13.9 (1.1–76.4)	14.7 (1.3–74.0)	13.5 (1.1–76.4)	
From first cell-free DNA collection	8.4 (0.3–33.0)	6.3 (0.8–33.0)	10.7 (0.3–24.7)	

Percentages are based on the patients with data available. Source data are provided as a Source Data file.

^a^Bonferroni-corrected threshold for significance: *p* = 0.0025.

^b^For patients who received treatment for their metastatic disease during the follow-up period.

As a comparator to our metastatic cohort, we performed targeted sequencing (median unique read depth of 1456×) on cfDNA from 39 patients initially diagnosed with local or locally advanced muscle-invasive bladder cancer (MIBC); samples were collected prior to curative-intent treatment. Twenty-one percent (8/39) of MIBC patients had ctDNA >1%, significantly lower than the 83% (59/71) observed in mUC patients with cfDNA collected prior to first-line systemic treatment for their metastatic disease (Fisher’s exact *p* < 0.00001; Supplementary Data 1 and 2 and Supplementary Fig. 3A, B). Recurrence-free survival was shorter in the eight MIBC patients with ctDNA >1% (hazard ratio (HR) 3.99, 95% confidence interval (CI) 1.13–14.1, *p* = 0.032; Supplementary Data 3 and Supplementary Fig. 3C, D), fitting with prior work suggesting that the presence of ctDNA at MIBC diagnosis is a poor prognostic factor^14^.

Pertinent to the design of future clinical efforts leveraging cfDNA, the abundance of ctDNA in mUC patients was impacted by collection timing in relation to systemic therapy. Samples collected after treatment initiation (but prior to clinical progression) had significantly lower ctDNA than those obtained prior to therapy or at progression (Kruskal–Wallis *p* = 2.4e − 07; Fig. 1c). Reductions in ctDNA levels coincided with the patient response (Supplementary Fig. 4). Prior surgical resection of the bladder was not associated with ctDNA fraction in patients with mUC (Fig. 1c). Importantly, for a biomarker source that must inform across a range of clinical scenarios, ctDNA abundance did not differ between patients subgrouped by sex or upper tract involvement (Fig. 1b). Young age (<60 years, approximately the 25th percentile) was modestly associated with an increased ctDNA fraction (median 30.5% versus 8.6% (60–75 years) and 7.3% (>75 years, 75th percentile); Kruskal–Wallis *p* = 0.04; Fig. 1b).

There are few clinical prognostic factors in mUC^16–18^. Encouragingly, we observed a significant relationship between ctDNA fraction below the first quartile and improved overall survival (OS) among 71 patients initiating first-line systemic therapy for metastatic disease (HR 3.15, 95% CI 1.32–7.48, *p* = 0.01; Fig. 1d and Supplementary Data 3). When evaluated in a multivariate model incorporating clinical factors (Eastern Cooperative Oncology Group performance status ≥ 2 and the presence of visceral metastases, individually or as a combined score^16^), ctDNA fraction was independently associated with OS (*n* = 65; HR 3.59, 95% CI 1.47–8.75 and HR 3.51, 95% CI 1.45–8.45, respectively; Supplementary Data 3).

### ctDNA reproduces the tumor tissue driver genome

**Fig. 2:**
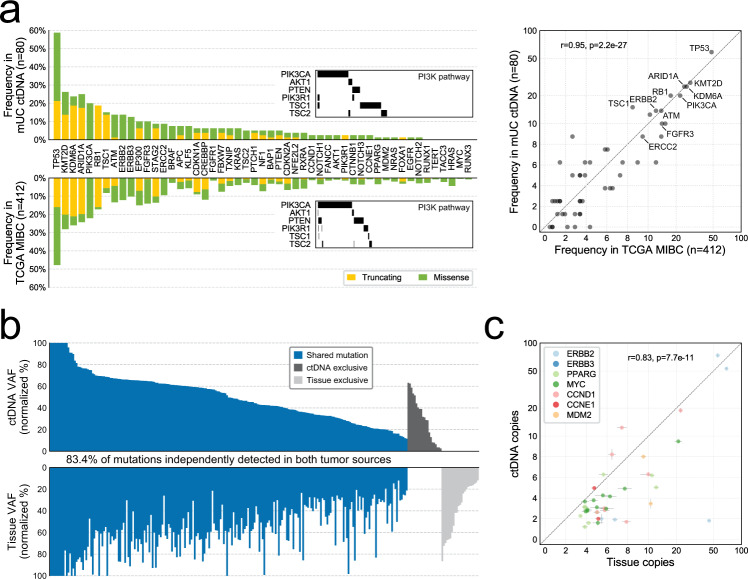
Comparison of circulating tumor DNA (ctDNA) to tumor tissue. **a** Gene mutation frequency and mutation type in metastatic urothelial carcinoma (mUC) ctDNA versus The Cancer Genome Atlas (TCGA) localized muscle-invasive bladder cancer (MIBC) cohort, across 50 driver genes on our targeted panel. TCGA information was obtained via cBioPortal. **b** Detection of protein-altering somatic mutations in ctDNA and patient-matched tumor tissue from 46 patients. Variant allele fractions (VAFs) for 265 mutations detected via targeted DNA-sequencing are normalized to tumor fraction estimates. **c** Correlation of gene copy number between ctDNA and tissue for seven commonly amplified oncogenes. Linear regression *p* value calculated for 38 amplification events across 27 patients (remaining 19/46 patients lacked amplifications in selected oncogenes). Data are presented as the exact gene copy number estimate (dot), +/− the 95% confidence interval (error bar) as calculated per gene from the coverage log ratio in samples with no evidence of cancer (tumor fraction = 0). Source data for (**a**) and (**c**) are provided as a Source Data file.

Remarkably, using ctDNA alone, we independently reconstructed the driver gene landscape of aggressive primary disease, as defined in The Cancer Genome Atlas (TCGA) analysis of muscle-invasive tissue^7^. Gene somatic alteration frequency, type of alteration, and even mutually exclusive relationships between driver events were paralleled in our metastatic ctDNA cohort (Fig. 2a, Supplementary Data 4, Supplementary Fig. 5, and Supplementary Note 1). Mutagenesis in bladder cancer is mediated, in part, by aberrant APOBEC activity^7,19^, and consistent with this, AID/APOBEC-associated tri-nucleotide mutational signatures 2 and 13 (16.3% and 20.9%, respectively) were abundant across the 35 cfDNA samples subjected to WES (Supplementary Data 5 and Supplementary Fig. 6).

To assess the similarity between genomic profiles derived from tissue versus ctDNA, we retrieved 94 patient-matched primary and/or metastatic tumor tissue specimens from 63 of 104 mUC patients and applied the same targeted sequencing approach (Fig. 1a and Supplementary Fig. 1c). The tissue tumor fraction, as estimated from targeted sequencing, ranged from 5 to 98.7% across the 95% (89/94) of samples with somatic mutations detected (Supplementary Data 1 and 2). Six tissue samples had tumor fractions below our detection thresholds via targeted sequencing, which was confirmed via WES (Supplementary Data 1 and Supplementary Fig. 2d). Importantly, patients with matched tissue did not differ from the remainder of the cohort in terms of their clinical characteristics, ctDNA fraction, TMB, or genomic landscape (Table 1 and Supplementary Fig. 7a).

To limit false-negative mutation calls in low tumor purity samples, we restricted comparison to 46 patients with tissue–ctDNA pairs where both samples had a sufficient tumor fraction to detect protein-altering somatic mutations (Supplementary Data 6 and Supplementary Fig. 7b). For each patient, we evaluated the highest ctDNA fraction sample and the most recent tissue sample (when multiple samples were available); 89% of the most recent tissue samples were from a muscle-invasive or metastatic lesion (Supplementary Fig. 1c). Across the 46 pairs, targeted sequencing detected 265 coding somatic mutations; 83.4% (221/265) were independently detected in both tumor sources, while 7.9% (21/265) and 8.7% (23/265) were detected in ctDNA and tissue only, respectively (Fig. 2b and Supplementary Data 7). All tissue–ctDNA pairs shared at least one mutation (Supplementary Fig. 8). For 43% (19/44) of mutations exclusive to tissue or ctDNA, at least three unique reads supporting the mutant allele were observed in the paired sample, indicating that higher depth sequencing could increase their independent detection. However, low sequencing coverage did not explain the majority of exclusive mutation calls (Supplementary Data 7), suggesting genuine differences in the subclonal composition between a single primary tumor focus and bulk ctDNA in mUC. Accordingly, subclonal mutations were less frequently shared between tissue–ctDNA pairs than clonal mutations (32/50 versus 189/215; Fisher’s exact *p* = 0.0002).

Although it is challenging to identify copy number changes from formalin-fixed paraffin-embedded (FFPE)-derived DNA and samples with low tumor purity, oncogene amplification was strongly correlated between tissue and matched ctDNA (*n* = 38 amplifications across 27 patients, Pearson’s *r* = 0.83, *p* = 7.7e − 11; Fig. 2c). No genes were enriched for mutation concordance or discordance after correction for multiple hypothesis testing (Supplementary Fig. 9).

### Mutation detection in ctDNA is consistent despite intra-patient heterogeneity

**Fig. 3:**
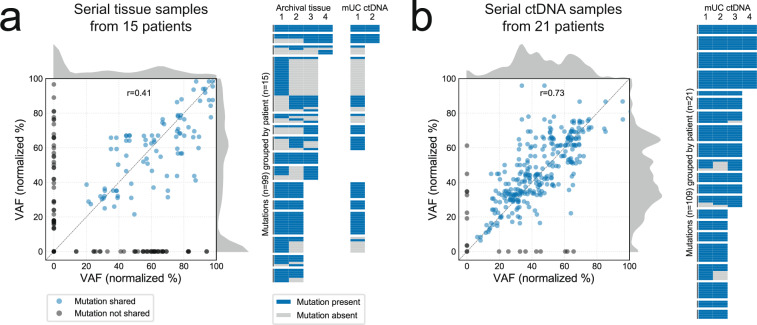
Temporal heterogeneity in tumor tissue and circulating tumor DNA (ctDNA). **a** Mutation detection across same-patient serial tissue samples. Correlation of somatic mutation variant allele fractions (VAFs) in paired tissue samples, with mutations not detected in one member of the pair (VAF = 0) shown in gray (left). Kernel density estimates show a peak in mutations detected exclusively in one sample. Each unique mutation detected in serial tissue is plotted as a row in the heatmap (right), along with their re-detection in ctDNA-positive samples (if available). **b** Mutation detection across same-patient serial ctDNA samples. Somatic mutation VAFs are strongly correlated (left), with few mutations not consistently detected (right). In both (**a**) and (**b**), VAFs are normalized to tumor purity, and those >100 (e.g., on amplified genes) are not shown. Source data are provided as a Source Data file.

Temporal heterogeneity was explored across serial tumor tissue samples collected over the course of disease progression in 15 patients (2–4 samples per patient, 42 total). Forty-five percent of mutations were independently detected across all asynchronous patient-matched tissue samples (Fig. 3a). Conversely, in 21 patients where serial ctDNA profiles could be compared (two to four samples per patient, 59 total), 90% of the mutations were independently called in all samples (Fisher exact *p* < 0.00001; Fig. 3b). Differences in sequencing depth (median 1040× in ctDNA versus 370× in tissue) did not account for the inferior concordance observed in tissue—12% (12/97) of mutations inconsistently called across serial tissue samples could be explained by insufficient coverage, versus 54% (7/13, all below 3% variant allele fraction) in serial ctDNA. Furthermore, although the median interval between ctDNA pairs was shorter than for tissue pairs (1.6 versus 4.9 months), serial tissue samples collected within 6 months had fewer shared mutations than ctDNA pairs within the same interval (50% versus 100% median concordance, Mann–Whitney *U* test *p* = 0.0008; Supplementary Fig. 10a). Tissue to tissue variability is likely partly explained by somatic clonal shifts that occur during progression from non-muscle-invasive to muscle-invasive disease^20^, and subclonal heterogeneity in multifocal and/or recurrent primary lesions. In line with this, mutation concordance of mUC ctDNA with non-muscle-invasive tissue was significantly lower than with muscle-invasive and metastatic tissue (50% versus 89% median concordance, Mann–Whitney *U* test *p* = 0.002; Supplementary Fig. 10b).

Primary MIBC is molecularly heterogeneous, and classifications based on transcriptome profiling are associated with differential patient prognosis and distinct classes of driver alterations^21^. We performed RNA-sequencing on 79 tissue samples, in parallel with targeted DNA-sequencing (Supplementary Data 1), and applied a consensus classification system that identifies six transcriptome subtypes (Supplementary Fig. 11)^22^. We noted a split between samples with luminal versus basal gene expression signatures, and expression of key genes was consistent with canonical datasets (muscle-invasive tissue from systemic treatment-naive patients), despite the classifier not being trained for use across our heterogeneous cohort that includes post-treated metastatic tissue and non-muscle-invasive samples (Supplementary Fig. 12). There was a non-significant trend for stroma-rich tissue samples to have lower tumor fractions than other consensus subtypes (55% versus 68% median tissue cancer fraction, Mann–Whitney *U* test *p* = 0.07; Supplementary Fig. 13a). Notably, the majority of metastatic tissue samples clustered in the stroma-rich subtype, and shifts to a stroma-rich subtype, were frequent among patients with serial tissue samples; post-treatment subtype shifts are consistent with the development of a scar-like phenotype^23^. Together, these data highlight the challenge of applying existing RNA subtyping models in late-stage disease where contemporary tissue samples are rarely treatment-naive (Supplementary Fig. 13b).

### ctDNA has advantages over tumor tissue for real-time genomic biomarker evaluation

**Fig. 4:**
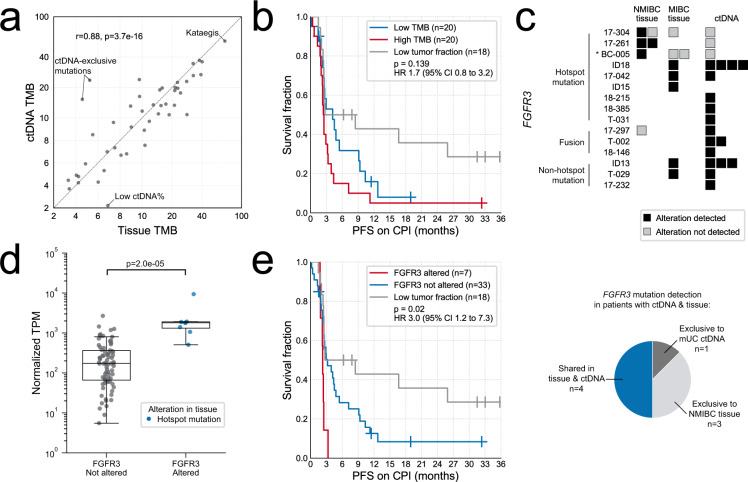
Tumor mutational burden (TMB) and *FGFR3* status evaluation in circulating tumor DNA (ctDNA) and tumor tissue. **a** TMB estimates from the highest ctDNA fraction sample and most recent tissue sample for 46 metastatic urothelial carcinoma (mUC) patients. *P* value calculated using linear regression. **b** Kaplan–Meier survival analyses for progression-free survival (PFS) in the subset of patients treated with immune checkpoint inhibitors (CPI), stratified by median TMB (12.4 mutations/Mb). Statistical significance was measured using Cox proportional hazards regression analysis; non-evaluable patients (those with insufficient ctDNA to detect protein-altering somatic mutations) were excluded from the survival regression. **c** Detection of alterations in *FGFR3*. Asterisks indicate TMB > 30 mutations/Mb. Samples with a tumor fraction of zero are not shown. NMIBC non-muscle-invasive bladder cancer, MIBC muscle-invasive bladder cancer. **d** *FGFR3* expression levels in eight tissue samples with activating alterations, compared to 78 tissue samples without *FGFR3*-activating alterations detected via targeted DNA-sequencing. No *FGFR3* rearrangements were detected in the tissue. *P* value calculated with two-sided Mann–Whitney *U* test. Boxplots are centered at the median, with the boxes spanning the first to third quartile, and minima and maxima extending to 1.5× IQR. TPM transcripts per million. **e** Kaplan–Meier survival analyses for PFS in the subset of patients treated with CPI, stratified by *FGFR3* alteration status. Statistical significance was measured using Cox proportional hazards regression analysis; patients with low tumor fractions (insufficient to detect protein-altering somatic mutations) were excluded from the survival regression. Source data for (**a**) and (**d**) are provided as a Source Data file.

In some cancers, tissue TMB is a biomarker for immunotherapy response. In mUC, tissue TMB alone does not appear to robustly associate with patient benefit, although it may still have utility as part of a biomarker suite^24^. We extrapolated somatic mutation counts from targeted sequencing of ctDNA to obtain estimates of genome-wide TMB ranging from 0.75 to 57.4 mutations/Mb, with a median TMB of 10.6 (Supplementary Data 1). Median TMB for muscle-invasive and metastatic tissue together was 11.1 (interquartile range (IQR) 7–19), comparable to muscle-invasive tissue analyzed with a similarly sized commercial panel^25^. TMB was similar between tissue–ctDNA pairs (*n* = 46, Pearson’s *r* = 0.88, *p* = 3.7e − 16; Fig. 4a and Supplementary Data 1). However, there were two cases with relatively high TMB in mUC ctDNA, but low in primary tissue (Fig. 4a). TMB correlation was also lower when comparing ctDNA to older tissue samples (*n* = 14, Pearson’s *r* = 0.53, *p* = 0.05). In exploratory biomarker analyses, we did not observe a relationship between ctDNA TMB and duration of response to immune checkpoint inhibition or platinum chemotherapy (Fig. 4b, Supplementary Data 3, and Supplementary Fig. 14a).

The pan-FGFR inhibitor erdafitinib recently received regulatory approval based on a 40% response rate in advanced patients with *FGFR* alterations detected by tissue profiling^3^. We detected somatic *FGFR3* mutations in ctDNA from eight patients with mUC (Fig. 4c). Importantly, these mutations were independently detected in all ctDNA-positive samples and patient-matched tissue. All *FGFR3* mutations had VAFs suggestive of truncal status (Supplementary Data 8). *FGFR3* mutations are enriched in non-muscle-invasive lesions relative to adjacent muscle-invasive disease^26^, and consistent with this, in three patients we observed *FGFR3* p.S249C mutations in non-muscle-invasive tissue that were not detected in later muscle-invasive tissue or mUC ctDNA samples (Fig. 4c). In a further three patients, breakpoints in ctDNA indicated the presence of activating *FGFR3* gene fusions (Fig. 4c, Supplementary Data 9, and Supplementary Fig. 14b). In total, 13.8% of the ctDNA-evaluable mUC cohort (11/80) exhibited *FGFR3* alterations likely to sensitize their tumors to erdafitinib. Tissue samples with activating *FGFR3* alterations demonstrated elevated *FGFR3* expression (Fig. 4d). Surprisingly, one *FGFR3-TACC3* fusion identified in ctDNA was not identified in earlier TURBT tissue (despite other shared genomic alterations); consistent with this, tissue *FGFR3* RNA expression was below the 25th percentile. In an exploratory subgroup analysis, we observed a modest association between *FGFR3* alteration and shorter progression-free survival (PFS) for patients receiving immunotherapy (*n* = 58; PD-1/PD-L1/CTLA-4, single agent or in combination) (Fig. 4e).

**Fig. 5:**
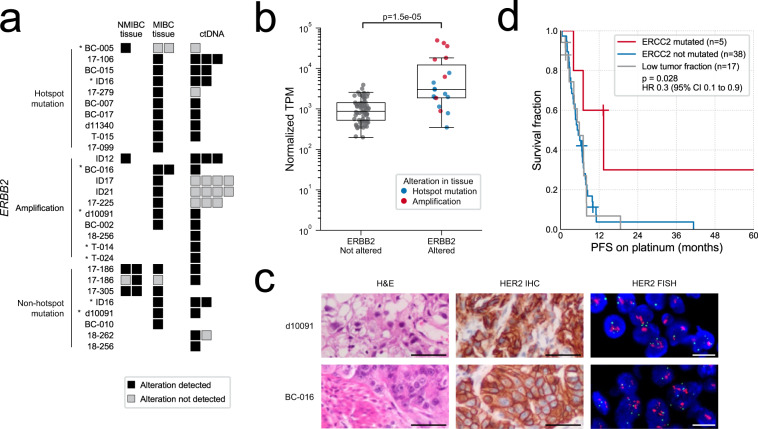
Evaluation of *ERBB2* and *ERCC2* status in circulating tumor DNA (ctDNA) and tumor tissue. **a** Detection of alterations in *ERBB2*. Asterisks indicate TMB > 30 mutations/Mb. Samples with a tumor fraction of zero are not shown. NMIBC non-muscle-invasive bladder cancer, MIBC muscle-invasive bladder cancer. **b** *ERBB2* expression levels in 19 tissue samples with activating alterations, compared to 67 tissue samples without *ERBB2*-activating alterations detected via targeted DNA-sequencing. *P* value calculated with two-sided Mann–Whitney *U* test. Boxplots are centered at the median, with the boxes spanning the first to third quartile, and minima and maxima extending to 1.5× IQR. TPM transcripts per million. **c** Tissue staining for *ERBB2* (HER2) amplifications detected in ctDNA: hematoxylin and eosin (H&E), and positive immunohistochemistry (IHC) and fluorescence in situ hybridization (FISH). IHC and FISH were performed once per patient specimen using a clinically validated test. Scale bars correspond to 50 µm (H&E and IHC) and 10 µm (FISH). **d** Kaplan–Meier survival analysis for progression-free survival (PFS) in the subset of patients treated with platinum chemotherapy, stratified by *ERCC2* mutation status. All but one mutation fell within a helicase domain. Statistical significance was measured using Cox proportional hazards regression analysis; patients with low tumor fractions (insufficient to detect protein-altering somatic mutations) were excluded from the survival regression. Source data for (**b**) are provided as a Source Data file.

A subset of primary bladder cancers demonstrate somatic alterations and/or protein overexpression of *ERBB2* (HER2). Clinical trials of HER2-targeted agents have been unsuccessful in mUC, highlighting the difficulty of optimal patient selection^27^. *ERBB2* mutations were detected in 13.8% of the ctDNA-evaluable mUC cohort (11/80), and gene amplification was detected in ctDNA from seven patients, two of whom harbored simultaneous *ERBB2* mutation (Fig. 5a). The absolute *ERBB2* copy number was ≥50 in three cases, enabling detection despite low ctDNA fractions (Supplementary Fig. 14c). Several tissue samples with activating *ERBB2* alterations detected by DNA-sequencing also exhibited elevated *ERBB2* expression (Fig. 5b). In two patients with *ERBB2* amplification detected in ctDNA, we confirmed gene amplification and protein overexpression via clinical-grade fluorescence in situ hybridization (FISH) and immunohistochemistry (IHC) in patient-matched primary tissue (Fig. 5c). For three patients, *ERBB2* amplification was identified in tissue but not in later ctDNA despite shared mutations; correspondingly, *ERBB2* intra-patient heterogeneity has been reported between primary tumors and lymph node metastases^28^. We considered the possibility of false-negative variant calls, but for both *FGFR3* and *ERBB2* alterations the majority of discordances did not appear to be due to insufficient tumor fraction or sequencing depth; in these samples, other alterations were identified in both tissue and ctDNA, and sequencing depth across the genes was generally sufficient to detect truncal variants given the corresponding tumor purity (Supplementary Data 8).

Mutations in the DNA damage repair gene *ERCC2* are associated with sensitivity to neoadjuvant cisplatin chemotherapy^29^. Somatic *ERCC2* mutations were detected in 8.8% of ctDNA-evaluable mUC patients (7/80), and in the platinum-treated subset (*n* = 60; adjuvant or palliative cisplatin/carboplatin in first or second line) were associated with improved PFS (Fig. 5d and Supplementary Data 3). Among the patients where homologous recombination repair (HRR) and mismatch repair genes were assessed in ctDNA (sequenced on the 60 gene panel), protein-altering mutations were common. However, deleterious truncating mutations were not accompanied by a second somatic alteration, suggesting passenger status in accordance with urothelial carcinoma as a non-BRCA-associated cancer (Supplementary Fig. 15a)^30^. In one patient, we detected biallelic *BRCA2* deletion via targeted and WES of ctDNA (Supplementary Fig. 15b); tri-nucleotide mutational signature analysis revealed HRR-defect-associated signature 3 as one of the predominant signatures in this patient (12%; Supplementary Data 5), similar to BRCA2-deficient metastatic prostate cancer.

## Discussion

Our study in a large mUC cohort benchmarks a blood-based “liquid” biopsy against patient-matched tumor tissue for identifying somatic alterations. We demonstrate that a liquid biopsy is sufficient to resolve contemporary driver gene events in ctDNA-positive patients, suggesting that future biomarker-driven protocols can leverage clinically practical blood draws for patient stratification. Importantly, the analysis of cfDNA in blood and urine has already promised utility in the context of early bladder cancer diagnosis and detection of metastatic relapse following cystectomy^14,31^. Therefore, considering our hypothesis-generating study, we posit that cfDNA profiling is poised for near-term clinical impact across the spectrum of aggressive bladder cancer.

In samples with sufficient ctDNA to detect protein-altering somatic mutations, driver gene status was highly consistent with metastatic lesions and primary muscle-invasive disease. However, there were notable discordances when comparing mUC ctDNA to older primary tissue specimens, particularly non-muscle-invasive foci. Furthermore, while bulk metastatic driver genotype in serial ctDNA was typically invariable, we observed frequent temporal heterogeneity in serial primary site tissues. Several prior tissue studies in bladder cancer have revealed intra-patient clonal heterogeneity and somatic evolution during disease progression^20,32^. Clonal shifts are well documented during localized bladder cancer invasion into the muscle-bed; for example, the common loss of *FGFR3* hotspot mutations^26^. Therefore, our data suggest that ctDNA provides a more representative snapshot of mUC disease genomics than a single archival primary tumor focus.

Primary bladder cancer has a high mutation rate and shows genome scars of aberrant APOBEC enzymatic activity^7,19,33^. Indeed, APOBEC-related mutational signatures were detectable here in mUC ctDNA. Mutation rates are elevated in late versus early-stage disease^34^, suggesting that mutational processes remain active during progression. As such, some intra-patient and intra-tumor heterogeneity is inevitable, and serial ctDNA-based surveys of the genome will reveal genetic drift and variable passenger events. However, genomic events that confer fitness advantages (such as driver mutations) are less likely to vary, which may explain the high consistency between serial ctDNA samples in our study (targeted sequencing), compared to a study of 32 patients where broader exome profiling suggested extensive intra-patient heterogeneity in advanced post-chemotherapy disease^32^. This concept is supported by a rapid autopsy study (seven patients) where clinically informative alterations tended to be shared between metastatic lesions regardless of exome-wide heterogeneity^35^. Driver events arising late in tumorigenesis, or those that confer context-dependent survival advantages, will be the exception; fitting this model, in our exploratory analysis we identified several examples where *FGFR3* or *ERBB2* alterations were absent from mUC ctDNA, despite their presence in primary lesions prior to clinical metastatic progression.

In this study, 15% of mUC patients had an estimated ctDNA fraction below 1% of total cfDNA. Low ctDNA did not appear to associate with distinct genome or transcriptome patterns in matched archival tissue (although, notably, no mUC patient with the luminal-papillary tissue subtype had a ctDNA fraction >30%, as this subtype is reported to be the least clinically aggressive^22^). Furthermore, while ctDNA fractions in other metastatic cancers are linked to patterns of disease burden such as visceral spread^9,36^, ctDNA abundance appeared independent of the presence of visceral metastases in mUC. Future studies should examine whether the burden of metastatic disease, as reflected by the number and size of measurable lesions, is associated with ctDNA fraction. Potential links between ctDNA fraction and transcriptomic subtype should also be further explored, especially since spatial and temporal heterogeneity in gene expression patterns are well documented^23^. Despite these vagaries, low or undetectable ctDNA should not constitute a complete “test fail” in mUC, since this patient subgroup had the best OS from initiation of first-line systemic therapy. Two prior studies (of 16 and 27 mUC patients, respectively) also noted prognostic trends for ctDNA abundance^37,38^, indicating potential value across a range of clinical scenarios and treatment regimens. Clearly, ctDNA fraction as a prognostic biomarker now requires prospective validation in mUC. However, given the relative lack of available prognostic factors in mUC, and the fact that ctDNA abundance was independent of known clinical prognosticators, we anticipate that cfDNA profiling could augment existing models for estimating patient life expectancy^39^. Such models are of particular importance given the recent expansion of therapeutic options for mUC^3,4,40^.

Several patients with low ctDNA had clinically relevant alterations identified by matched tissue analysis, suggesting that complete reliance on a liquid biopsy may miss opportunities for genomics-driven care. It is possible to consider a model where tumor tissue DNA profiling could be considered as a fallback in these patients, especially given that their relatively good prognosis provides time to source archival blocks or perform a fresh metastatic biopsy. Of course, tissue-based analyses have an associated failure rate since not all biopsies or surgical resections yield tumor-derived DNA of sufficient quality for sequencing. In tissue landscape studies, it is uncommon to report the broader denominator of patients without available tumor tissue or with “test fails.” We note that only 57% of muscle-invasive bladder cancer cystectomy cases passed quality control for inclusion in TCGA 2014 data freeze^41^.

We set a high threshold for ctDNA characterization, several fold above the theoretical limit of ctDNA detection^42^. In low ctDNA samples, we frequently observed rare reads supporting mutations identified in matched tissue, suggesting that deeper sequencing approaches would increase detection sensitivity. However, ultra-deep sequencing is prohibitively expensive when performed across large portions of the genome. Furthermore, elderly and sick individuals often have somatic clones present in their circulation that are independent of their primary cancer diagnoses^43–45^. These clones will compromise specificity for ctDNA detection unless focusing solely on disease-restricted hotspot mutations (e.g., in *FGFR3*) or mutations defined from prior tumor tissue testing. As such, different commercial tests show poor concordance for mutations with allele fractions below 1% when comparing patient-matched samples^46–49^.

We have demonstrated that ctDNA profiling can identify previously proposed biomarkers for therapy response in bladder cancer, including alterations in *FGFR3*, *ERCC2*, and *ERBB2*, and TMB. A cost-effective and minimally invasive method for their identification will enable patient stratification in biomarker-driven clinical trials, as well as real-world implementation of precision oncology. However, the intricate relationship between the driver genome, RNA subtype, and clinical history in bladder cancer means that comprehensive studies are still required to dissect the underlying biology of tumor aggression and treatment response^4^. The prediction of response to checkpoint inhibitors is a notable example; TMB in isolation is a poor biomarker^50–52^. Furthermore, while *FGFR3* alteration is strongly associated with response to FGFR inhibitors, there are conflicting reports of links to checkpoint inhibitor resistance^53^. In our exploratory analysis, we observed a weak association between *FGFR3* alteration and shorter PFS in mUC patients receiving checkpoint inhibitors, but this is unlikely to be clinically useful in isolation. Likewise, the genomic context must be taken into account; despite harboring activating alterations, in many tumors *ERBB2* is probably not a relevant therapeutic target (e.g., subclonal mutations in hypermutated tumors).

Monitoring ctDNA serially can reveal treatment-related alterations to the somatic genome, aiding in optimizing the sequencing of therapies. Moreover, change in ctDNA abundance may be useful as a biomarker of response^38,54^; fitting with this, we found that ctDNA fractions were decreased in patients receiving treatment, relative to pre-therapy initiation or progression samples. Across same-patient metastatic samples, we observed minimal temporal heterogeneity, suggesting that historical treatments for mUC (platinum chemotherapy and immunotherapy) do not drastically re-shape the driver genome. Likewise, the genomic landscape in the metastatic setting is highly similar to localized, muscle-invasive primary tissue^7^, suggesting that these aggressive primary tumors may reflect metastatic disease in situ. This lack of evolution is in contrast with other cancers where driver alterations become enriched during metastatic progression, and in bladder cancer, treatment may alternatively impact mutation signatures, immune markers, and RNA subtypes. Emerging targeted therapies (anti-FGFR and Nectin-4) are likely to change this paradigm, since treatment resistance will require the tumor to alter or lose the target.

Our study is limited by the heterogeneous (real-world) nature of the cohort and lack of pre-specified sample collection time points. Tissue samples represent those available from routine clinical practice, much like the tissue profiled in recent trials of targeted therapies in mUC. Despite at times small subgroup numbers, our retrospective analyses provide compelling results that warrant validation in future studies of mUC ctDNA genomics.

## Methods

### Patient cohort

Between December 2014 and November 2018, 192 whole blood samples were collected from 104 patients. Our retrospective cohort included patients with cancer of the urinary bladder and/or upper urinary tract (any histologic variant) with at least one distant metastatic lesion (M1). Blood was collected from patients at any stage of their treatment for metastatic disease. Samples were categorized as “prior to treatment initiation” if blood was drawn from patients prior to receipt of systemic therapy (any line) for their metastatic disease. If the patient had received ≥1 day of systemic therapy at the time of collection, the sample was categorized as “after treatment initiation”; this included blood collected after a patient had completed a course of treatment (e.g., post six cycles of platinum chemotherapy), but prior to disease progression. Finally, samples collected at the time of documented progression (clinical or radiological) were categorized as “progression.” Note that since the date of disease progression was determined by the treating physician, and often back-dated to the day of imaging results, progression blood samples were not always collected on the same day (range relative to documented progression: 0–15 days, median 5 days). Common systemic therapies received included platinum chemotherapy and anti-PD-1/PD-L1 agents, although treatment was not exclusive to these and trial patients were not excluded. Patient clinical records were reviewed for the availability of archival tissue specimens. Where possible, tissue was retrieved and submitted for pathology review to identify tumor-rich foci. The sampling method (e.g., single core, multiple cores, scrolls) was dependent on the tissue available and pathologist preference. In total, 94 patient-matched tissue specimens were retrieved from 63 of the 104 patients. The majority of tissue samples were FFPE archival specimens (90/94), while the remaining four were fresh frozen tissue. We also identified pre-treatment cfDNA samples from 40 patients with localized MIBC, collected consecutively to our liquid biobanking program between June 2017 and March 2020. Study approval was granted by the University of British Columbia Clinical Research Ethics Board, the Ethics Committee of Ghent University Hospital, and the Ethics Commission of the Medical Faculty of the Eberhard-Karls-University Tübingen and University Hospital Tübingen. The study was conducted in accordance with the Declaration of Helsinki, and written informed consent was obtained from all participants prior to enrollment.

### Sample processing, library preparation, and sequencing

Whole blood was collected in 4 × 6 mL EDTA or 2 × 9 mL Streck Cell-Free DNA BCT® tubes. Blood in EDTA tubes was centrifuged at 1600 r.c.f. and 4 °C for 2 × 10 min within 1–2 h of collection. For Streck BCT tubes, the time from blood collection to processing was 0–5 days, with a median of two. Blood collected in Streck tubes was kept at room temperature prior to and during processing where samples were centrifuged at 1600 r.c.f for 15 min, after which plasma was transferred to a new tube and spun for an additional 10 min at 3200 r.c.f. Aliquots of buffy coat (leukocytes for germline DNA; gDNA) and plasma were obtained simultaneously and stored at −80 °C prior to DNA extraction. For samples collected at Ghent University Hospital, Bimetra Biobank protocols were followed for sample processing with plasma obtained during peripheral blood mononuclear cell (PBMC) isolation^55^.

Plasma cfDNA was extracted from up to 6 mL of input with the QIAGEN Circulating Nucleic Acids Kit, and quantified with the Quantus Fluorometer and QuantiFluor ONE dsDNA system or Qubit 2.0 Fluorometer and Qubit dsDNA HS Assay Kit. Matched gDNA was extracted from the buffy coat fraction/PBMC using the QIAGEN DNeasy Blood and Tissue Kit, or the Promega Maxwell® RSC Blood DNA Kit and Promega Maxwell® RSC Instrument. Extracted gDNA was quantified with a NanoDrop spectrophotometer. For archival FFPE tissue samples, DNA was extracted from cores or sections with the Covaris truXTRAC FFPE DNA Kit, or the Promega Maxwell RSC DNA FFPE Kit and Maxwell RSC system. DNA isolated from tissue was quantified as per cfDNA. RNA was extracted from FFPE tissue cores or sections with the Maxwell® RSC RNA FFPE Kit, and the Qubit™ HS Assay Kit and Agilent Bioanalyzer RNA Nano were utilized for quantity and integrity determination.

For all samples (cfDNA, gDNA, and tissue), we applied an established targeted DNA-sequencing strategy utilizing custom Roche NimbleGen SeqCap EZ Choice capture panels, modified by the inclusion of 4-bp molecular barcodes to the index sequence for some cfDNA libraries^13,54^. Both capture panels covered the exonic regions of a shared set of bladder cancer driver genes, chosen such that 98% of TCGA primary muscle-invasive tumors would have a nonsynonymous somatic mutation in at least one of the included genes. Final enriched library pools were sequenced on Illumina MiSeq (2 × 300 bp), NextSeq (2 × 150 bp), or HiSeq 2500 (2 × 125 bp) instruments. Select cfDNA samples with ctDNA fractions exceeding 25% (as determined from analysis of targeted sequencing data) subsequently underwent WES, together with matched gDNA. WES was performed using libraries previously prepared for targeted sequencing and following the identical protocols described for custom targeted sequencing, but instead utilizing the Roche Nimblegen SeqCap EZ MedExome Kit. For tissue-derived RNA, strand-specific ribo-depleted libraries were prepared and sequenced on a HiSeq 2500 (2 × 75 bp) by the BC Cancer Genome Sciences Centre (Vancouver, Canada).

### Analysis of sequencing data

Alignment and analysis of DNA-sequencing data were performed utilizing an established pipeline^9,13,54,56^. Somatic mutations were required to be supported by a minimum of eight mutant allele reads, with a minimum VAF of 1% in ctDNA and 8% in tissue. All somatic mutation calls were filtered against patient-matched gDNA, as well as the background error rate, in addition to meeting thresholds for mapping quality and read-end proximity. Our filters automatically remove putative tumor mutations that have significant read support in matched gDNA (i.e., the white blood cell compartment): we require mutations to have a VAF >3× that of the matched gDNA. This strategy serves to remove germline variants, and somatic variants related to hematopoietic stem cell clonal expansion. However, due to higher sequencing depth in cfDNA compared to matched gDNA, some low VAF mutations related to clonal hematopoiesis may not be filtered. Therefore, we manually reviewed all mutations and excluded three variants in genes linked to hematopoietic stem cell clonal expansion^57^ based on elevated gDNA VAF (Supplementary Data 4). For identification of silent mutations in the WES data, we required a 10% VAF at minimum, and that the VAF was at least 50× higher than the same-loci background rate and 10× higher than in the matched normal sample. We also required an average mapping quality >30 for reads supporting the mutation, and a read-end proximity score >25 (calculated as the average distance of the mutant allele from the nearest read end, among reads that support the mutation).

Comparison of gene alteration type and frequency to TCGA analysis of muscle-invasive bladder cancer tissue^7^ was performed using data obtained via cBioPortal. For mutual exclusivity analysis (Supplementary Note 1), TCGA data were downloaded from FireBrowse.

Tri-nucleotide signature weights were derived from WES data using a Python implementation of the deconstructSigs algorithm (v1.47), and COSMIC mutational signatures (v2)^33,58^. A minimum of 50 somatic mutations per sample was required for mutational signature analysis.

Gene expression levels were quantified using Kallisto 0.45.0^59^ with Ensembl v95 gene annotations and bias correction enabled. Transcript- and gene-level abundances were calculated using Tximport 1.10.1. Estimated counts were scaled using the average transcript length over samples and then to the library size. Normalized gene counts were calculated using DESeq2 1.22.2 using the median ratio method. The consensus molecular classification described by the Bladder Cancer Molecular Taxonomy Group was used to assign tumors in our cohort into six consensus messenger RNA (mRNA) subtypes: basal/squamous, luminal papillary, luminal non-specified, luminal unstable, stroma-rich, and neuroendocrine-like^22^. Likewise, we classified tumors based on the five subtypes reported by TCGA: basal squamous, luminal papillary, luminal, luminal infiltrated, and neuronal^7^. For both classifiers, the model was centroid-based and classifications were generated using provided code^22^. The stromal signature was calculated from the average expression of eight stromal-associated genes (*ACTG2*, *CNN1*, *MYH11*, *MFAP4*, *PGM5*, *FLNC*, *ACTC1*, and *DES*). The cell-cycle signature is the average of the E2F targets and G2M checkpoint signatures from the Molecular Signatures Database (MSigDB) hallmark gene set collection^60^. The immune190 signature scores were generated using the median of 190 immune-associated genes^61^.

### Estimation of tumor fraction and TMB

For cfDNA and tissue samples subjected to targeted sequencing, the fraction of cancer DNA was estimated based on the highest VAF among autosomal somatic mutations as tumor fraction = 2/(1/VAF + 1), conservatively assuming a loss of heterozygosity, since copy number changes are not readily detectable when tumor fraction is low. To deal with stochastic variation in observed variant allele read counts, we modeled the variant read count as arising from a binomial distribution, and conservatively calculated what the true VAF would be if the highest observed VAF was a 95% quantile outlier^9,13^. The somatic mutations utilized for estimation of tumor fraction are shown in Supplementary Data 2. All chromosome 9 mutations were excluded from tumor fraction estimation due to frequent copy neutral loss of heterozygosity, as were *TERT* promoter mutations due to low sequencing depth. Somatic mutations in copy number amplified regions were also excluded. Our limits for tumor fraction estimation were ~2% in ctDNA, and ~15% in tissue (as determined by the conservative somatic mutation calling thresholds requiring 1% VAF for ctDNA and 8% for tissue). While 88/104 patients had evidence of ctDNA, we excluded eight of those patients from downstream analyses due to a lack of protein-altering somatic mutation calls.

Mutation clonality versus subclonality was approximated based on purity-normalized VAFs to account for variable tumor fractions between samples (Supplementary Data 7–8). Subclonal mutations were defined as those with a VAF < 25% of the tumor fraction^62,63^; we applied this conservative threshold to account for the difficulty in accurately estimating cancer cell fractions from targeted sequencing data (where WES tools incorporating allelic copy number and ploidy estimation are not applicable).

A copy number-based approach was utilized for ctDNA fraction estimation from WES data; models testing different ctDNA fractions and diploid level log ratios were manually fitted to the genome-wide copy number data, and candidate models were rejected if any genes had a negative copy number. Some samples had a tumor fraction too low for accurate quantification-based on copy number (generally <20%), or highly complex copy number profiles (aneuploid and/or complicated by subclonality), and thus did not have models fit.

TMB (mutations per Mb) was calculated taking into account the number of genomic positions with sufficient coverage to detect a mutation with the same VAF. Specifically, for each sample TMB was determined by summing the input from each somatic mutation detected in a sample, using the formula 1/*B*_1_ + ⋯ + 1/*B*_*m*_, where *B*_*m*_ was the number of genomic sites with sequencing depth equal to or higher than required (≥8 mutant allele reads/VAF_*m*_) for detecting mutation *m*.

### Statistical analysis

All Mann–Whitney *U* tests performed were two-sided. Pearson’s *r* values and associated *p* values were calculated via linear regression.

Survival analysis was performed using Python 3.7.4 with lifelines v0.22.6^64^. Patients with low tumor fractions (no protein-altering somatic mutations detected) were excluded when fitting the Cox proportional hazard models given that the status of the genomic variable in question could not be determined. For the MIBC patients, RFS was calculated as the time from pre-treatment cfDNA collection to disease recurrence. In the mUC setting, OS was defined as either the time from first cfDNA collection to death or date of metastatic diagnosis to death. PFS was calculated as the time from treatment initiation to documented clinical or radiological progression, or death. Patients without documented events were censored at the date of the last follow-up.

### Reporting summary

Further information on experimental design is available in the Nature Research Reporting Summary linked to this paper.

## Supplementary information

Supplementary Information

Description of Additional Supplementary Files

Supplementary Data 1-9

Reporting summary

## Data Availability

All de-identified targeted and whole-exome DNA-sequencing data and RNA-sequencing data have been deposited in the European Genome-phenome Archive (EGA) database under the accession code EGAS00001004615 and is available under standard EGA controlled release. The TCGA data referenced [10.1016/j.cell.2017.09.007] are available in public repositories from the cBioPortal [https://www.cbioportal.org] and FireBrowse [http://firebrowse.org] websites. The MSigDB gene sets referenced [10.1016/j.cels.2015.12.004] are available from the GSEA website [https://www.gsea-msigdb.org/gsea/msigdb]. All the other data supporting the findings of this study are available within the article and its Supplementary information/data files, and from the corresponding author upon reasonable request. Source data are provided with this paper.

## Source data

Source Data

## Code availability

Custom computer code utilized for analysis is available on Github at https://github.com/annalam/muc-manuscript-code.
