# Supplementary material for: Plasma ctDNA is a tumor tissue surrogate and enables clinical-genomic stratification of metastatic bladder cancer
Source: Nat Commun. 2021 Jan 8;12:184. doi: 10.1038/s41467-020-20493-6 (PMC7794518; doi:10.1038/s41467-020-20493-6)
Supplement: Supplementary file 1 — Supplementary Information [file 41467_2020_20493_MOESM1_ESM.pdf]

Supplementary Fig. 1. Summary of the 104 metastatic urothelial carcinoma (mUC) patients, and their tissue and cell-free DNA (cfDNA) samples analyzed in this study. **a)** Patient characteristics (top) and samples per patient (bottom). Met. at Dx = metastatic at diagnosis; ECOG = Eastern Cooperative Oncology Group performance status. **b)** Clinical context of the cfDNA collections by the line of systemic therapy (Rx) for mUC, and timing of collection in relation to on-going treatment (prior to initiation, after initiation but prior to clinical progression, or at time of progression). CPI = immune checkpoint inhibitor. **c)** Tissue stage and source for the sequenced samples (left), and the stage of each patient's most recent sample (right). Primary tissue samples with uncertain muscle-invasive status are listed as unknown primary.

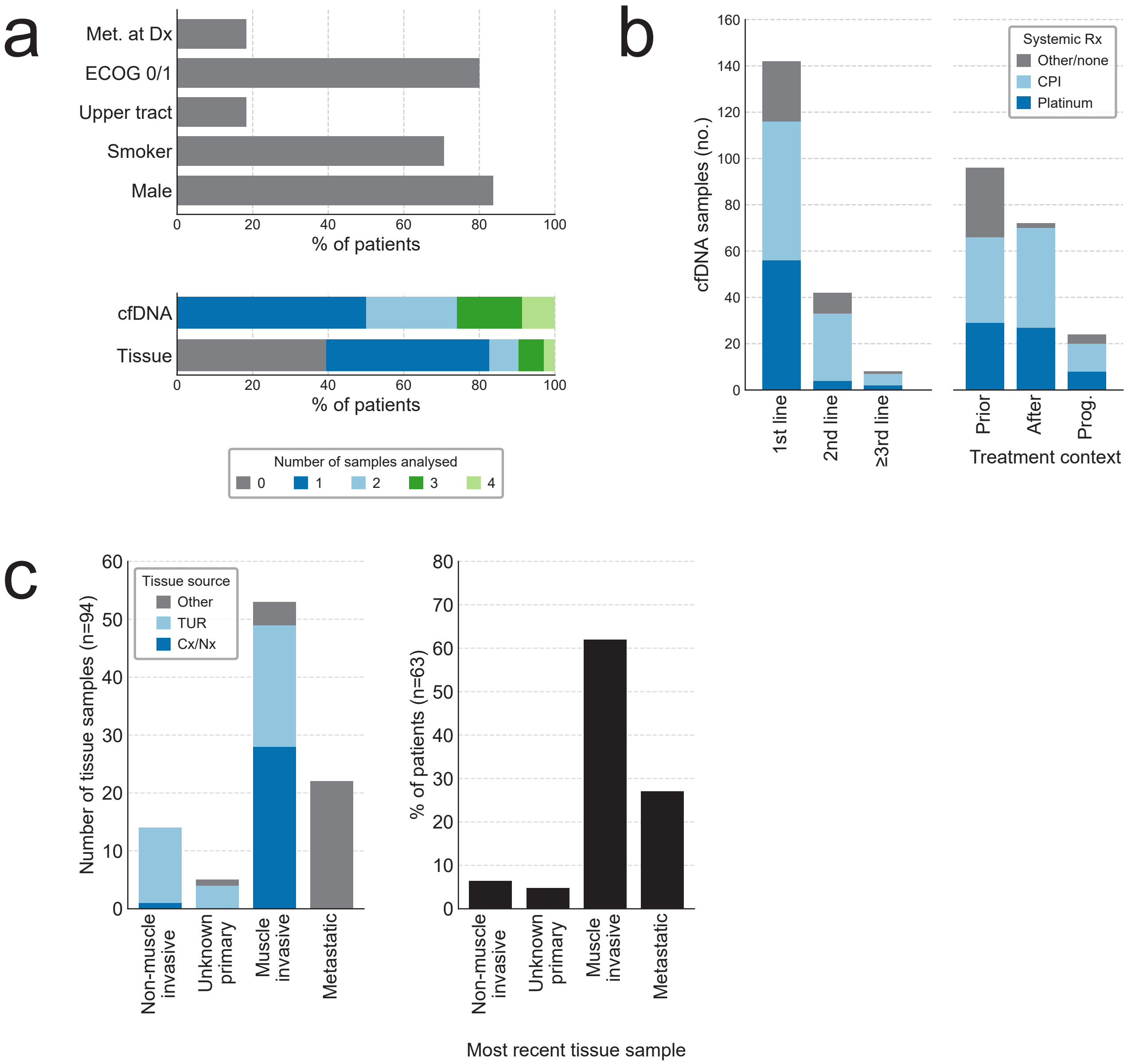

Supplementary Fig. 2. Estimation of tumor fraction. **a)** Determination of circulating tumor DNA fraction (ctDNA%, grey bar) from mutations detected via targeted DNA sequencing. The highest ctDNA% sample from 88 ctDNA-positive patients is shown, with the variant allele fraction (VAF) of each somatic mutation represented as a dot; mutations in black were excluded from the estimation (see Methods). **b)** Correlation of ctDNA% in 31 patient-matched serial plasma sample pairs (samples collected during treatment were excluded). P value calculated using linear regression. **c)** Targeted versus whole-exome sequencing (WES) tumor fraction (cancer %) estimates for 30 samples. P value calculated using linear regression. **d)** Allele fractions of somatic mutations (black dots) detected in the 49 tumor samples subjected to WES. VAF kernel density estimates are shown in gray. Samples are sorted by tumor fraction (as estimated from targeted sequencing), with n=35 ctDNA samples on the left and n=14 tissue samples on the right. Lines indicate the expected allele fractions of somatic mutations with and without somatic LOH.

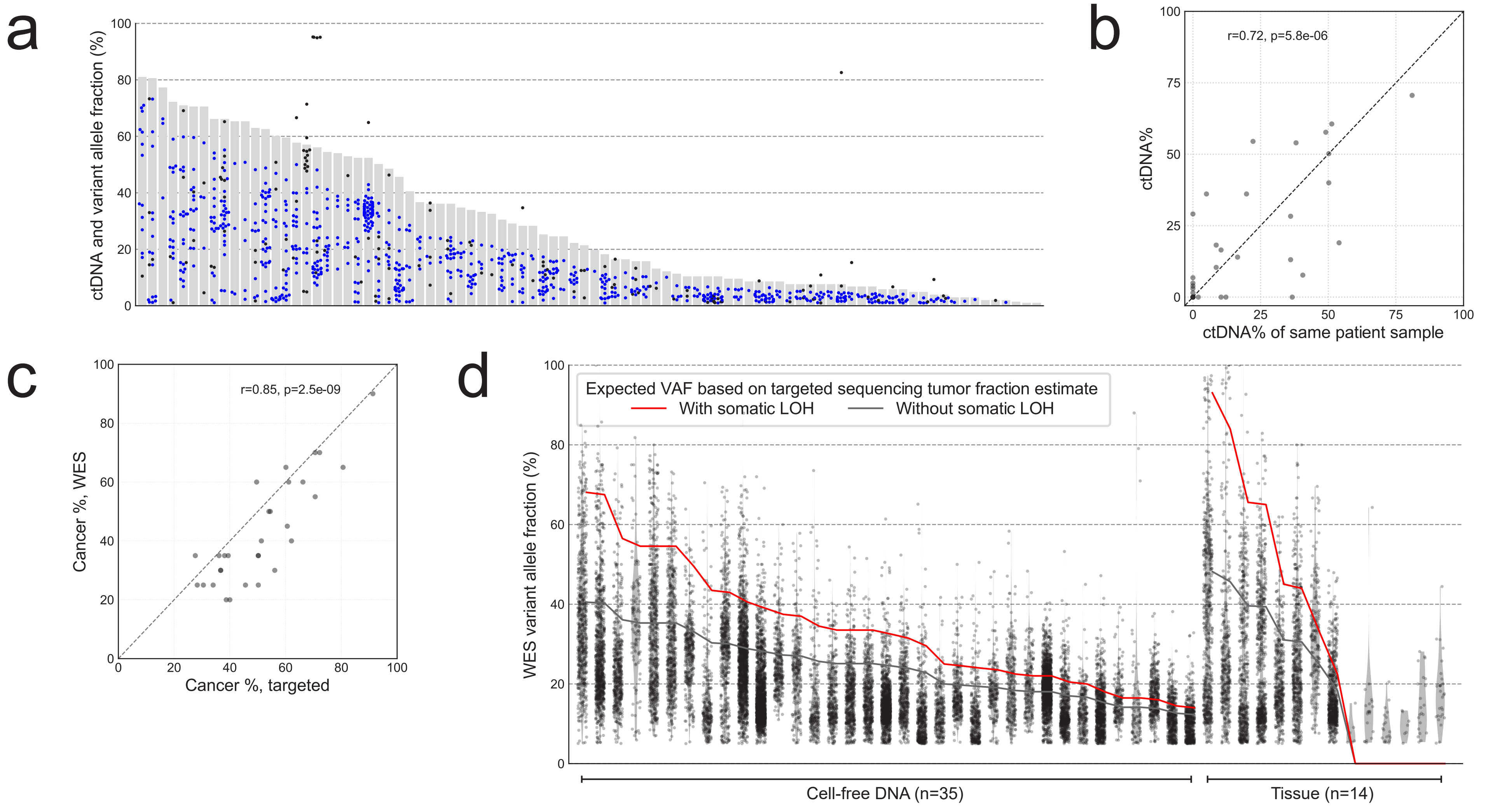

Supplementary Fig. 3. Circulating tumor DNA (ctDNA) abundance in muscle-invasive bladder cancer (MIBC). **a)** Comparison of ctDNA abundance in pre-treatment cell-free DNA (cfDNA) samples from patients with local or locally advanced MIBC versus metastatic urothelial carcinoma (mUC). P value calculated with two-sided Mann-Whitney U test. Boxplots are centered at the median, with the boxes spanning the first to third quartile, and minima and maxima extending to 1.5x IQR. **b)** The landscape of somatic alterations detected in ctDNA from MIBC patients. The top panel provides ctDNA fraction (ctDNA%) estimates, while the oncoprint integrates protein-altering somatic mutations and copy number results across altered genes on our targeted sequencing panel. **c)** Kaplan-Meier survival analysis for recurrence-free survival (RFS) in 35 MIBC patients with cfDNA collected prior to curative-intent treatment. Statistical significance was measured using Cox proportional hazards regression analysis. **d)** Clinical (cT/N) and pathological (pT/N) stage for 39 MIBC patients, highlighting ctDNA% does not strongly correlate with staging prior to (cT/N) or at (pT/N) cystectomy/nephroureterectomy.

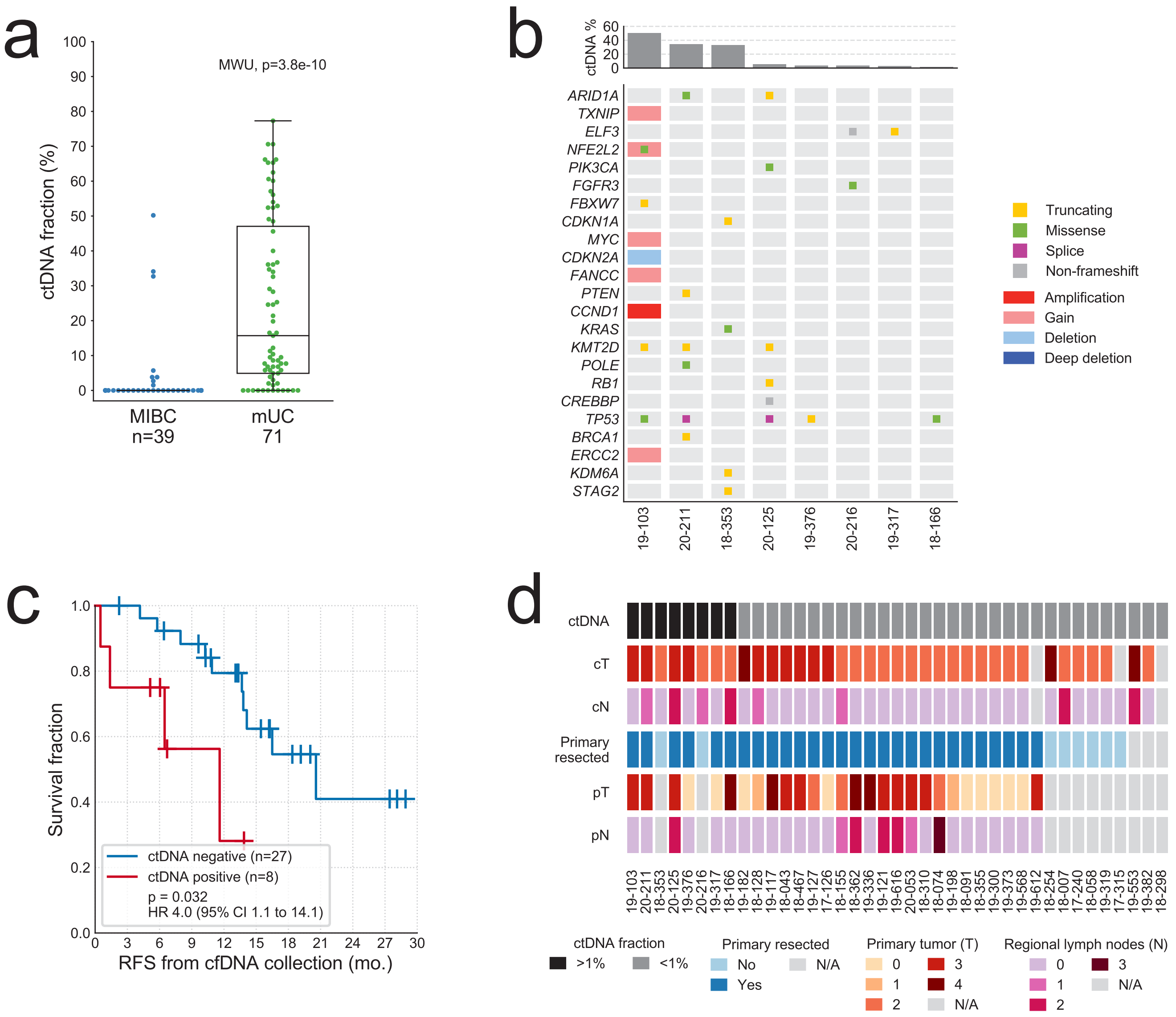

Supplementary Fig. 4. Change in circulating tumor DNA (ctDNA) abundance and association with treatment response in metastatic urothelial carcinoma (mUC). Twelve patients are represented who had blood collected pre-treatment and during or shortly after treatment, with at least one collection being ctDNA-positive; patients were also required to have CT scan records available clearly documenting response. Timing of collections, scans, and treatment duration are shown for each patient (left), alongside the absolute change in ctDNA fraction (right) from the pre-treatment to the follow-up sample. Arrow indicates direction of ctDNA% change.

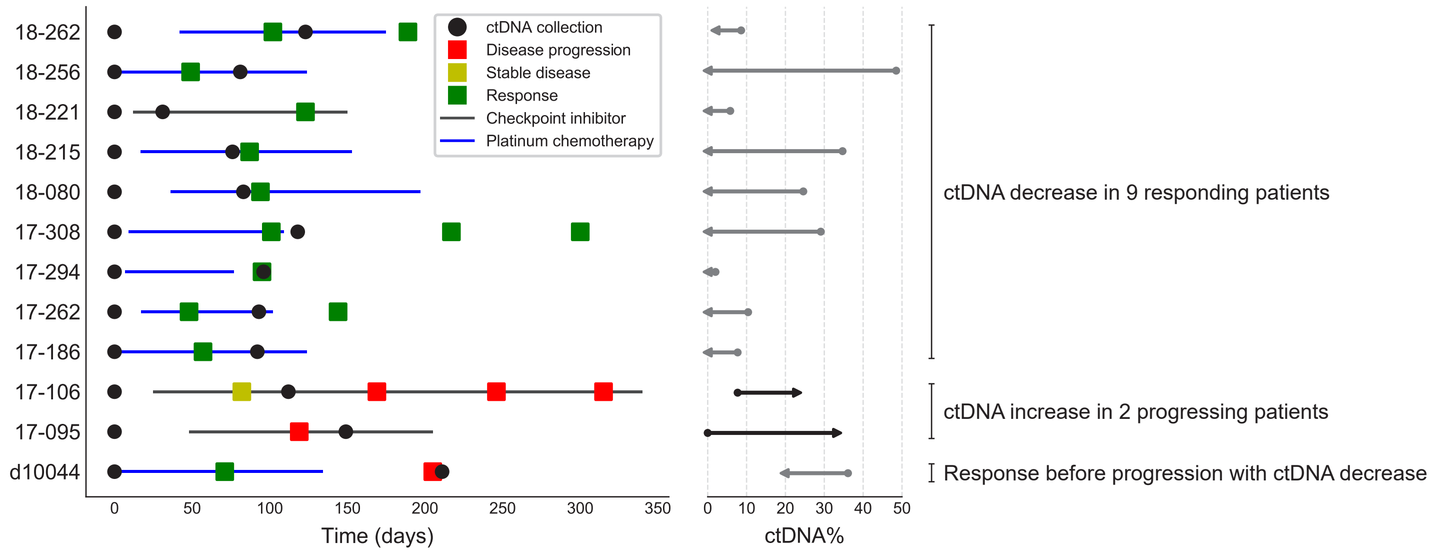

Supplementary Fig. 5. The landscape of somatic alterations detected in circulating tumor DNA (ctDNA) from metastatic urothelial carcinoma patients. Depicted is the highest ctDNA fraction (ctDNA%) sample from each patient with at least one protein-altering somatic mutation (n=80). The top panels provide tumor mutational burden (TMB) and ctDNA% estimates, while the oncoprint integrates mutation, copy number, and chromosomal rearrangement results across the genes on our targeted sequencing panel. Mutation counts per gene are summarized in the right-hand panel.

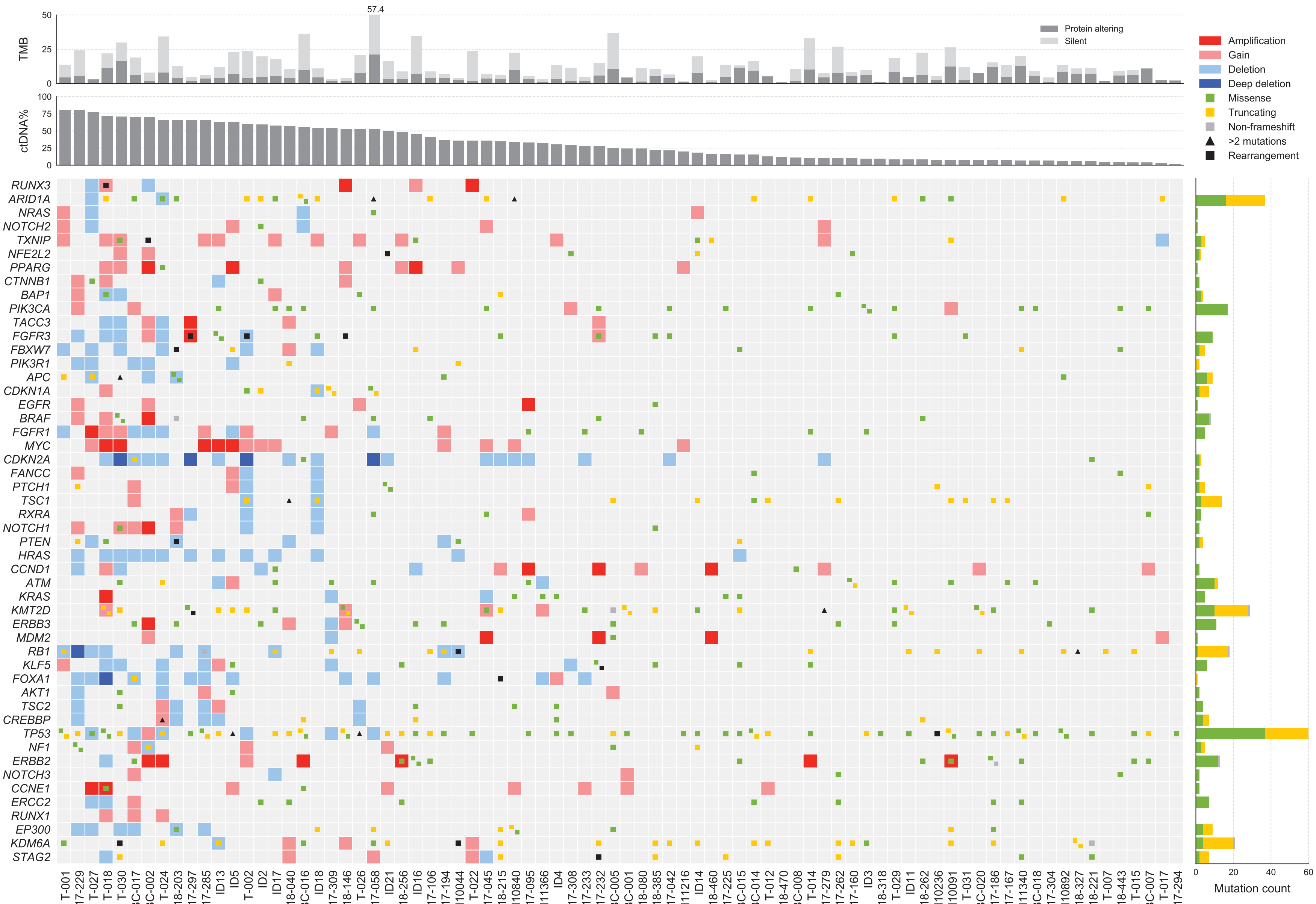

Supplementary Fig. 6. Tri-nucleotide signature analysis in whole-exome sequenced ctDNA from metastatic urothelial carcinoma (mUC), and metastatic castration-resistant prostate cancer (mCRPC) patients (from Annala *et al.*, Cancer Discovery 2018) analyzed in the same manner. For robust signature assignment, samples are restricted to those with a mutation count >50. AID/APOBEC-associated signatures (2 and 13) are predominant in mUC, and rare in mCRPC.

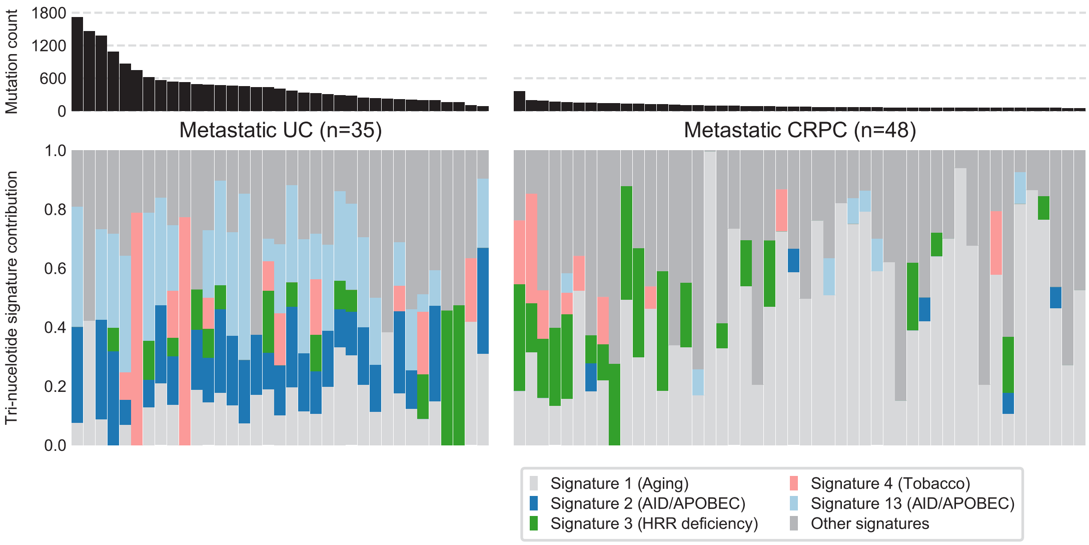

Supplementary Fig. 7. Similarity across cohort subsets. **a)** Comparison of circulating tumor DNA (ctDNA) fraction, tumor mutational burden (TMB) and gene mutation frequency in ctDNA between patients with matched tissue and those without tissue retrieved. P value calculated with two-sided Mann-Whitney U test. **b)** TMB and gene mutation frequency in tissue, between patients with tissue-ctDNA pairs included for comparison, versus those excluded based on low ctDNA fractions. Two additional patients with sufficient ctDNA were excluded due to an insufficient tumor fraction in their tissue sample (not shown). In both a and b, mutation frequencies are based on protein-altering somatic mutations, and TMB and gene mutation frequency analyses are limited to the subset of patients with protein-altering somatic mutations detected. All boxplots are centered at the median, with the box spanning the first to third quartile, and minima and maxima extending to 1.5x IQR. Note that the color scheme in the bar plots is the same as the box plots, with blue representing the mutation frequency in patients with matched tissue and green representing those without tissue retrieved.

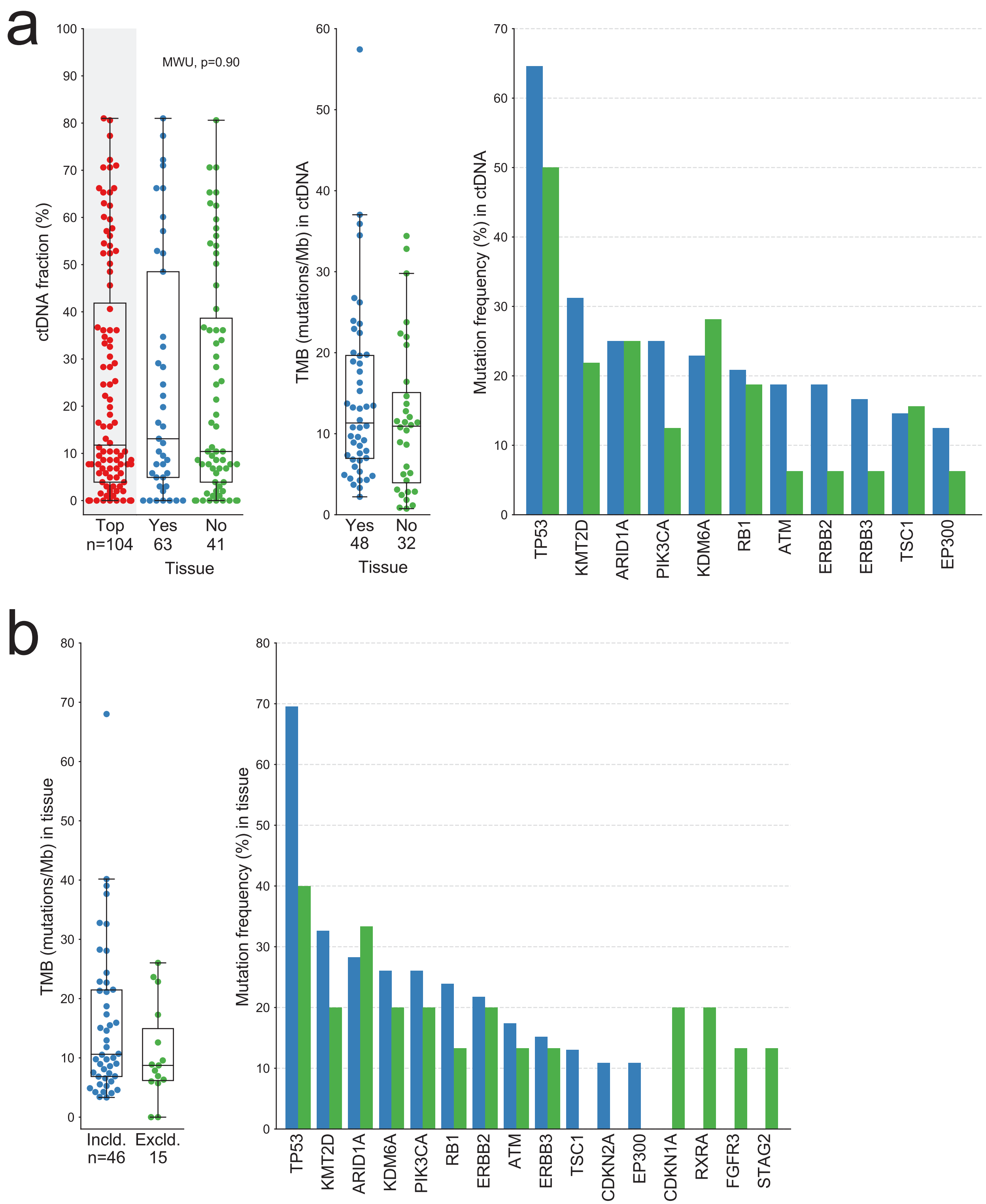

Supplementary Fig. 8. Detection of somatic mutations in 46 patient-matched circulating tumor DNA (ctDNA) and tissue pairs. Percent of protein-altering somatic mutations that were independently detected in both tumor sources (shared), versus those exclusive to ctDNA or tissue, is shown in the top panel for each patient. Time elapsed between sample collections and tissue stage are shown in the middle heatmap. In the main panel, results per gene per patient are vertically split with ctDNA (left) and tissue (right) shown in each box. Horizontal splits indicate multiple mutations.

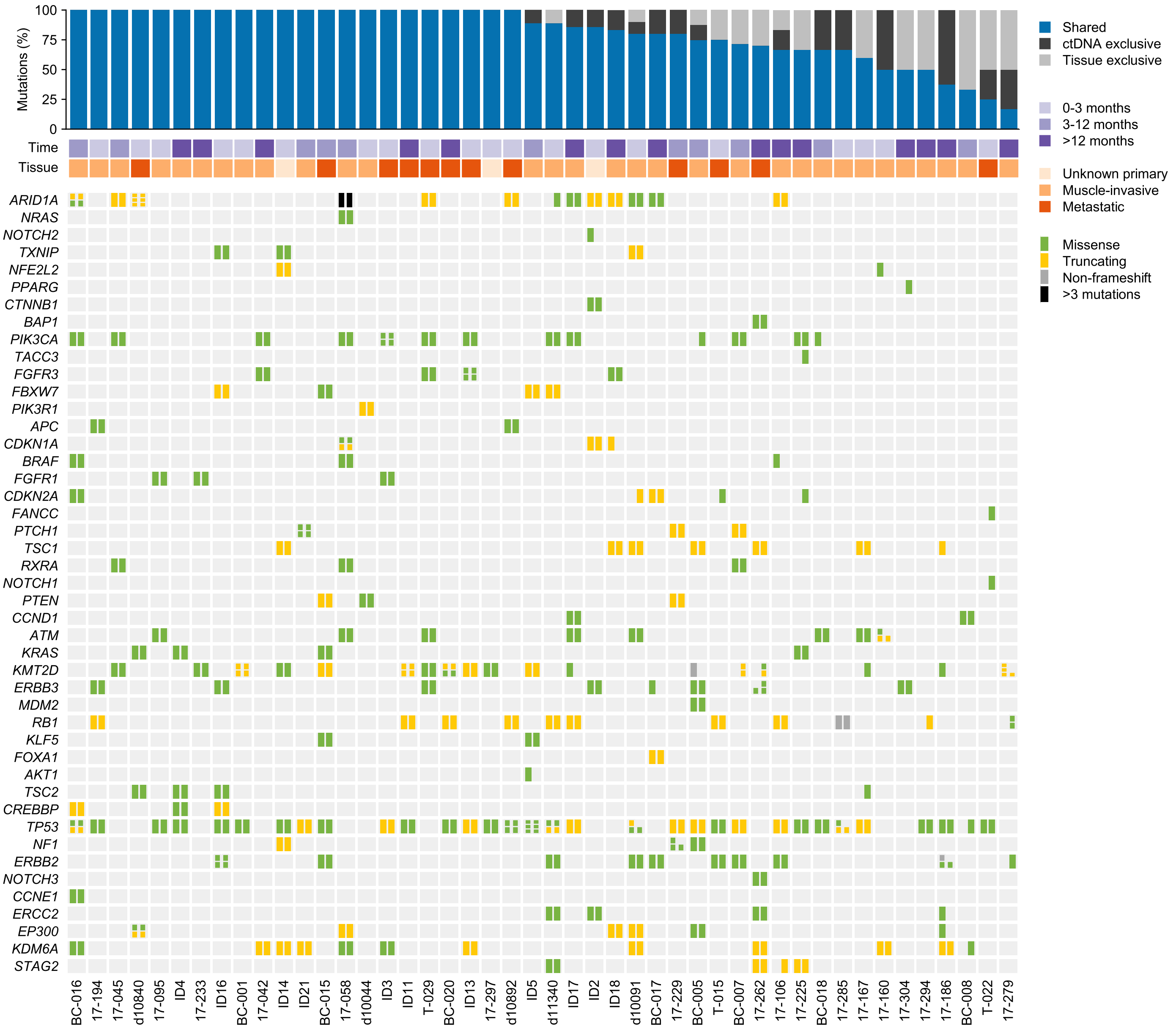

Supplementary Fig. 9. Detection of protein-altering somatic mutations in 46 patient-matched circulating tumor DNA (ctDNA) and tissue pairs. The top two panels highlight the tumor fraction of the highest ctDNA fraction sample and the most recent tissue sample, for which results are included in the main panel. The dotted lines indicate the median tumor fractions (for all sequenced samples with tumor fraction >0). Mutations per gene per patient are colored based on whether they were independently detected in each of the paired samples, or exclusively in one sample. Angled splits indicate multiple mutations.

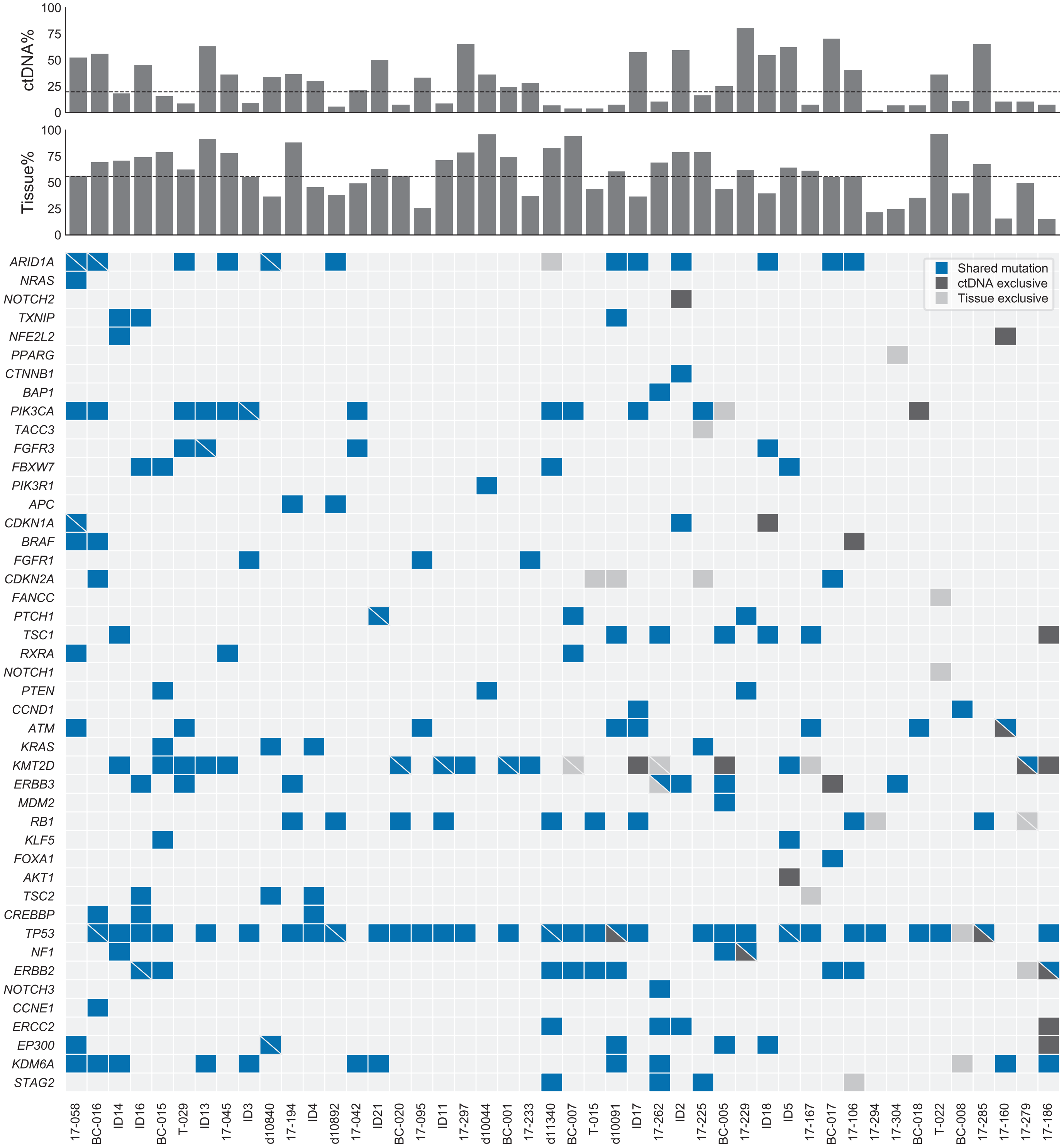

Supplementary Fig. 10. Somatic mutation detection in same-patient tissue and circulating tumor DNA (ctDNA) samples. **a)** Impact of time elapsed between collections on mutation concordance of patient-matched serial tissue (grey) versus serial ctDNA (red) samples. **b)** Impact of tumor invasiveness on mutation concordance of tissue with ctDNA. Concordance is based on each sample in comparison to the highest ctDNA fraction sample from that patient. In both a and b, error bars represent the standard error of the mean and p values are derived from Mann-Whitney U tests.

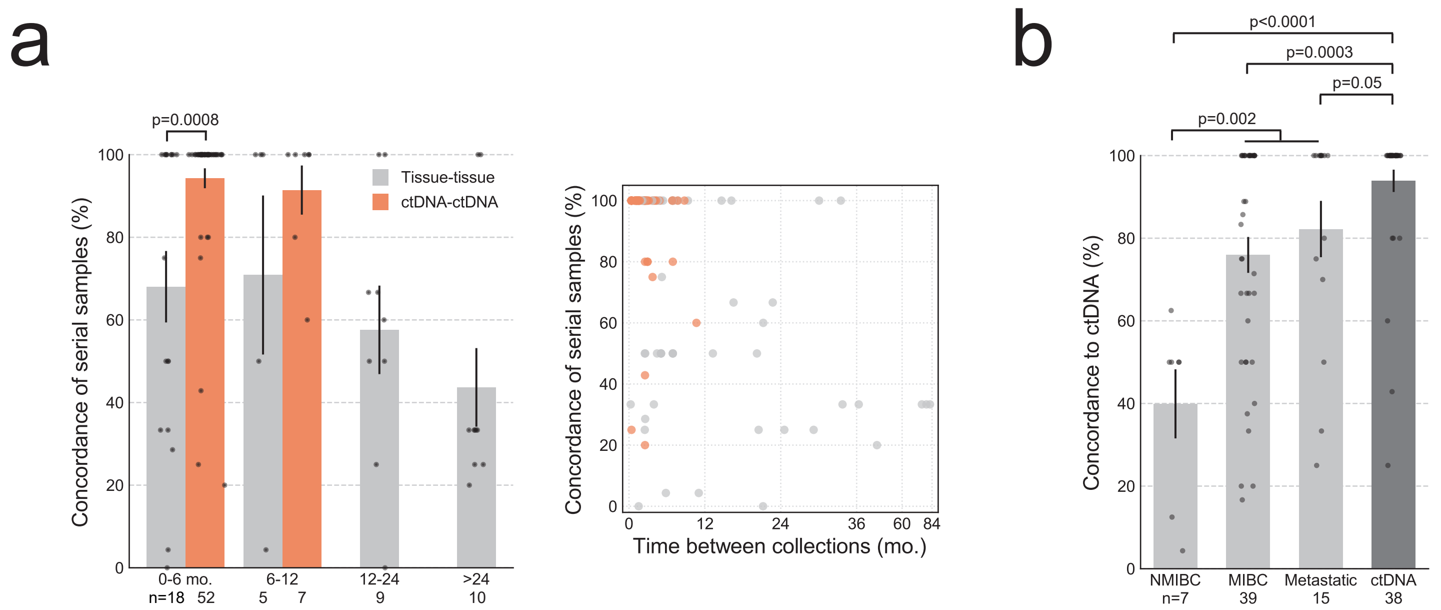

Supplementary Fig. 11. Gene expression subtyping and association with genomic alterations. **a)** Patients (columns) by Consensus classifier subtype and expression of key genes, in the most recent tissue sample that was subjected to RNA sequencing. Below, tissue tumor fraction and highest circulating tumor DNA (ctDNA) fraction for each patient, followed by results from targeted DNA sequencing of patient-matched ctDNA, or tissue in ctDNA-negative patients (represented sample indicated beneath barplot). Note, only amplifications and deep deletions are shown. NMIBC = non-muscle invasive bladder cancer; MIBC = muscle-invasive bladder cancer. **b)** Combined genomic alterations in each Consensus classfier subtype for the most recent tissue sample that was subjected to RNA sequencing per patient.

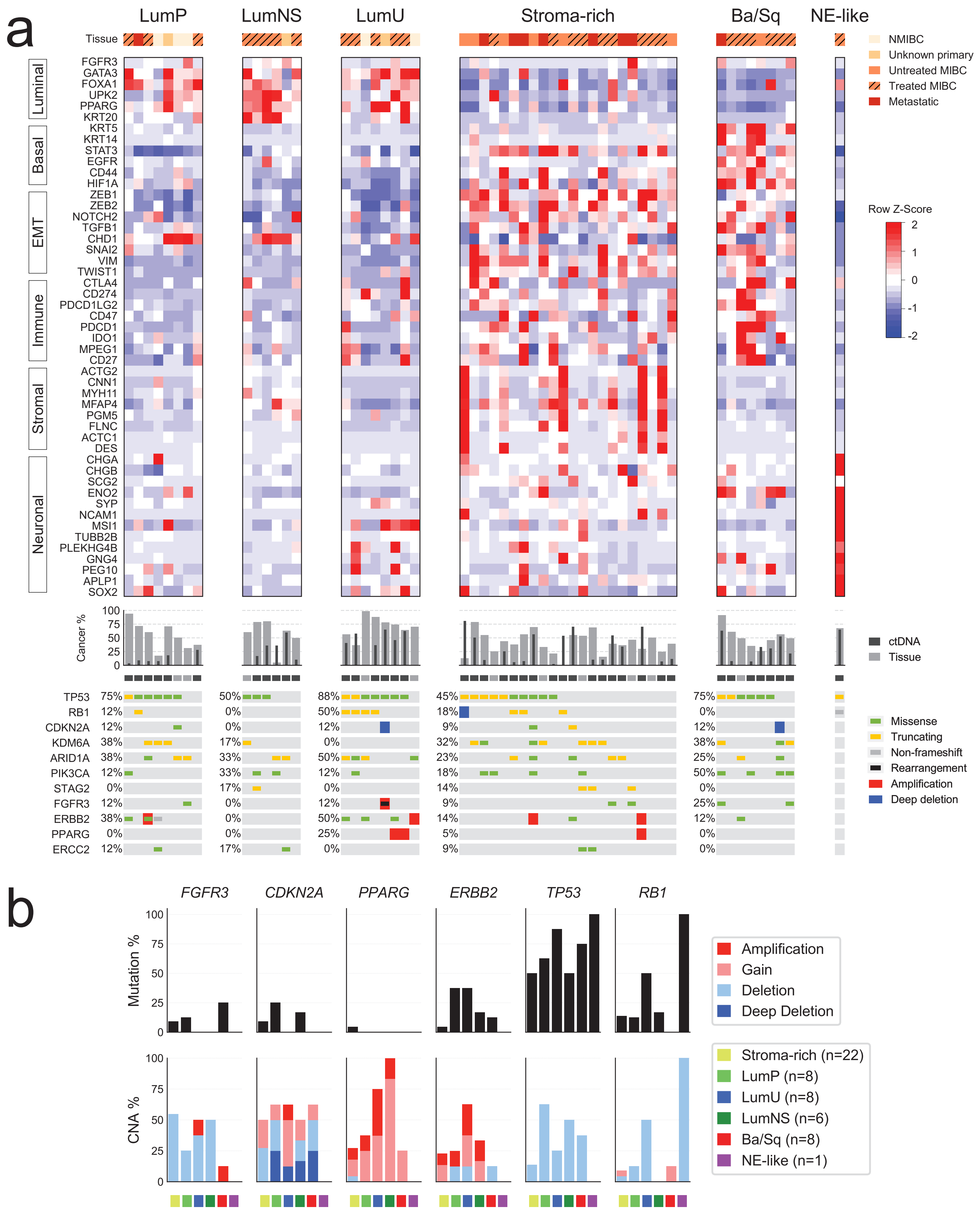

Supplementary Fig. 12. Expression of key genes and signatures in 82 tissue samples, stratified by molecular subtype as determined using the Consensus classifier. Gene expression in transcripts per million (TPM) is shown in the top six plots. Stromal, immune190, and cell cycle signatures are depicted in the bottom three plots. Kruskal-Wallis tests were used to generate p values. All boxplots are centered at the median, with the box spanning the first to third quartile, and minima and maxima extending to 1.5x IQR.

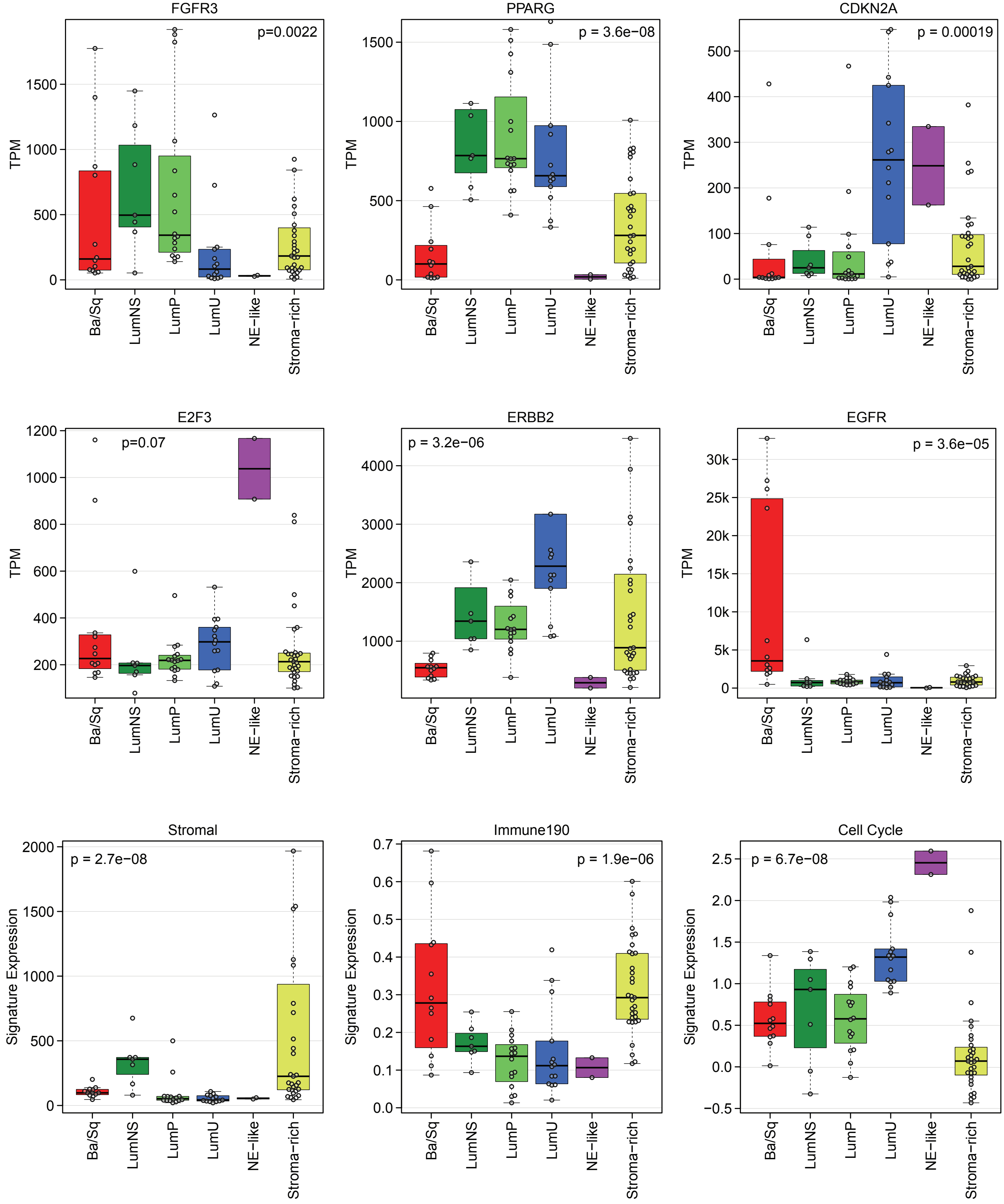

Supplementary Fig. 13. RNA molecular subtyping of tumor tissue. **a)** Tissue tumor fraction (of the same sample; top panel) and ctDNA fraction (highest per patient; bottom panel) for tumors stratified by molecular subtype using the Consensus (white background; LumP, LumNS, LumU, Stroma-rich, Ba/Sq, NE-like) and TCGA (grey background; Luminal papillary, Luminal infiltrated, Luminal, Basal squamous, Neuronal) classifiers. Kruskal-Wallis tests were applied to examine tumor fractions between subtype classifications and generate p values. Boxplots are centered at the median, with the boxes spanning the first to third quartile, and minima and maxima extending to 1.5x IQR. **b)** Consensus molecular subtypes of serially collected patient tissue samples, separated by disease state. Post-treatment muscle-invasive (MIBC) tissue samples are indicated with a hashed box. Samples from each patient are ordered left to right from earliest to most recent. NMIBC = non-muscle invasive bladder cancer.

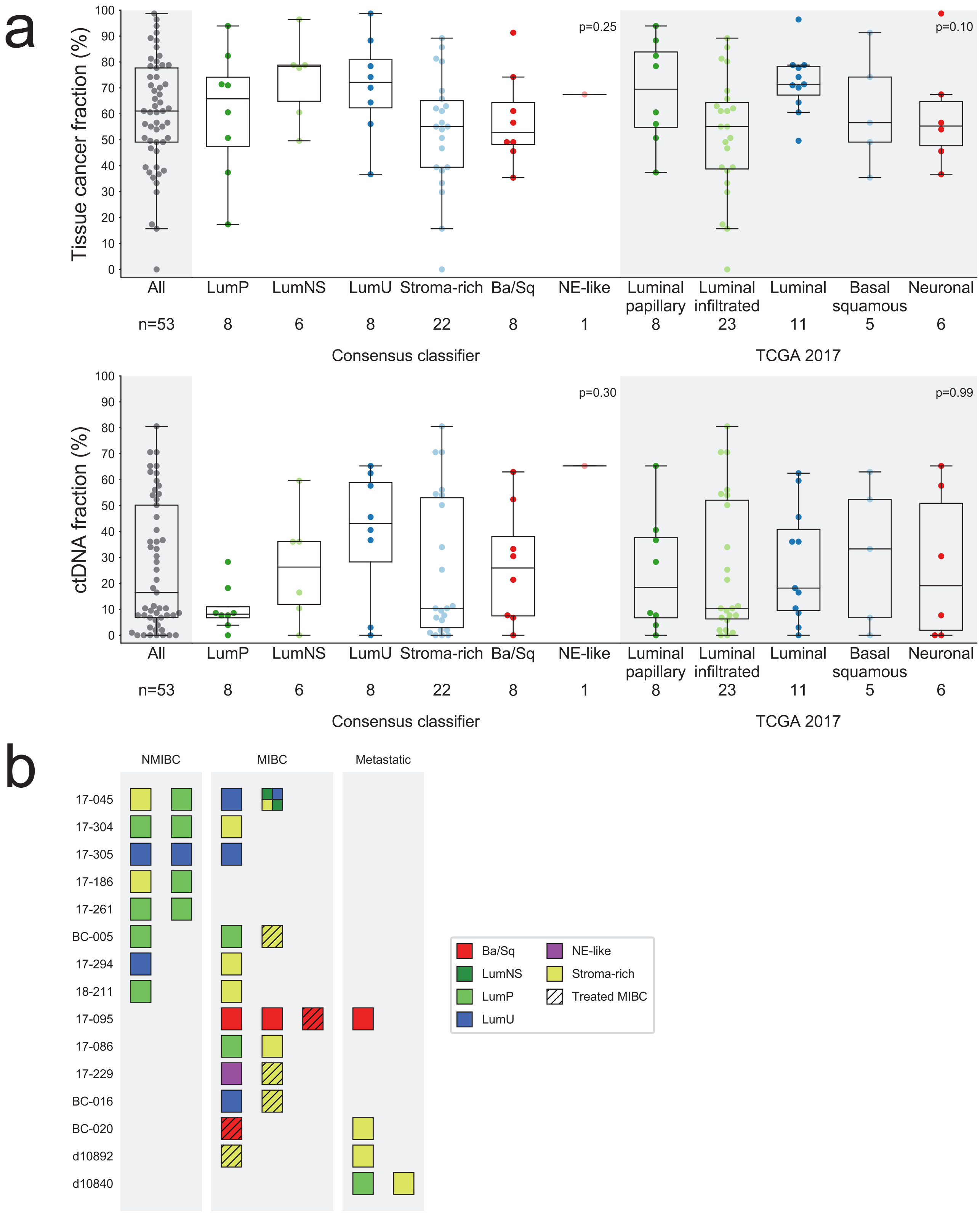

Supplementary Fig. 14. Biomarker detection in circulating tumor DNA (ctDNA). **a)** Swimmer plot of progression-free survival for 58 patients treated with immune checkpoint inhibitors. Bars are colored by tumor mutational burden (TMB), as estimated from the highest ctDNA fraction sample per patient. **b)** *FGFR3* gene fusions identified in ctDNA. Diagramed are the predicted chimeric in-frame *FGFR3-TACC3* and *FGFR3-ADD1* proteins, with a representative breakpoint-overlapping DNA sequencing read below. aa = amino acid. **c)** Detection of *ERBB2* gene amplification. The highest ctDNA fraction sample from each patient (n=104) is represented by a dot. Gene amplification calls (coverage logratio above 0.7) are highlighted in red. Curved blue lines represent the coverage logratio values corresponding to 5, 10, and 50 copy gains of *ERBB2*, as they relate to ctDNA fraction.

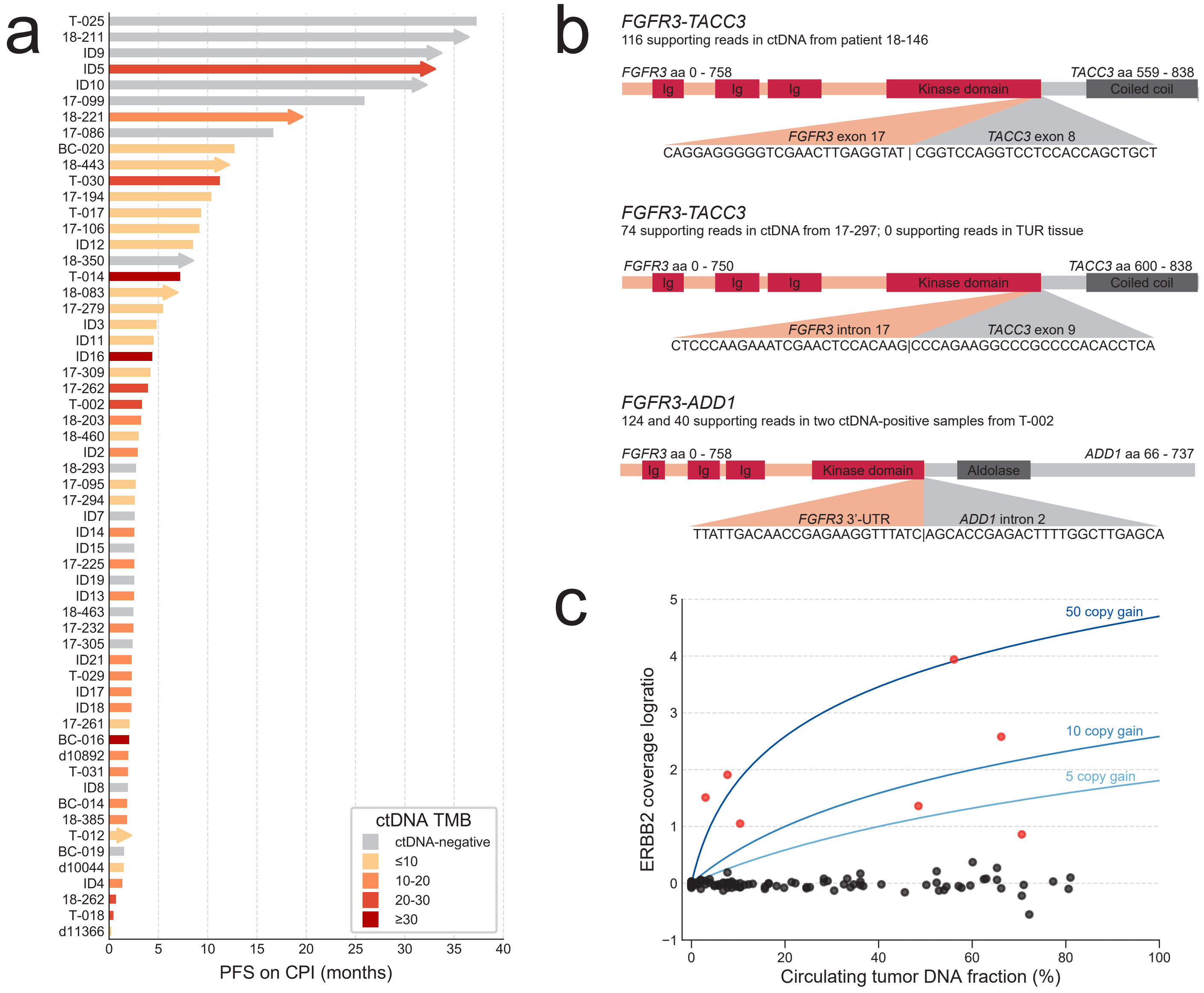

Supplementary Fig. 15. Homologous recombination repair (HRR) gene alterations in metastatic urothelial carcinoma (mUC). **a)** Evidence for loss of heterozygosity in HRR genes. Bar plot (left) depicts the allele fraction of each truncating HRR mutation detected in circulating tumor DNA (ctDNA), tissue, and/or germline DNA. The scatterplot (right) represents that GC-bias corrected coverage logratio of each gene relative to a median reference. Note that for patient 18-215 (*BAP1*), two separate mutations are shown (germline and somatic). An HRR mutation was not detected in 18-203, however, the coverage logratio indicates a bi-allelic deletion. ctDNA% = ctDNA fraction; tissue% = tissue tumor fraction. **b)** Copy number profile from patient 18-203 highlighting the *BRCA2* homozygous deletion. The coverage logratio violin plot shows the landscape of copy number alterations across 60 genes on the targeted panel. Copy number gain is depicted in light red, amplification in dark red, mono-allelic deletion in light blue, and bi-allelic deletion in dark blue.

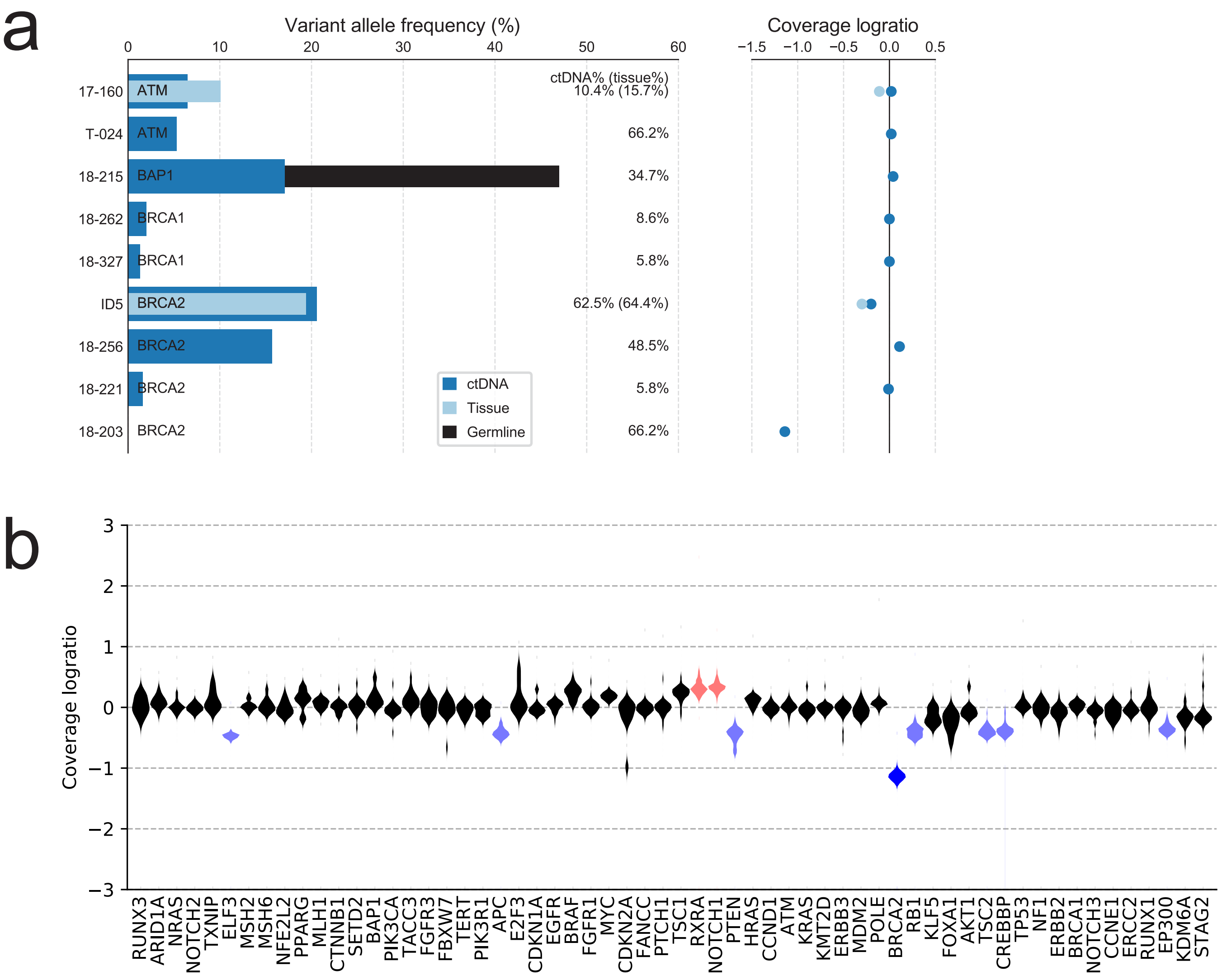

Supplementary Note 1

Mutual exclusivity

From mUC ctDNA, we observed a trend toward mutual exclusivity for mutations in the PI3K pathway, supportive of functional redundancy. We confirmed this trend by performing mutual exclusivity testing with DISCOVER [1] in TCGA BLCA data downloaded from FireBrowse (version 2016_01_28; groupwise test p = 0.027). Mutations in *FGFR3* and RAS family genes were also mutually exclusive in both our mUC cohort and primary muscle-invasive disease (pairwise combining *HRAS*, *KRAS* and *NRAS* p = 0.036; groupwise test p = 0.016), consistent with prior work in primary tissue [2]. For all comparisons, the raw mutation annotation file (n = 395), excluding all non-synonymous mutations, was used to generate the background matrix and the impurity method was employed for DISCOVER groupwise tests [1].

Allelic imbalance

We observed recurrent allelic imbalance across *RXRA* hotspot mutations related to copy neutral loss of heterozygosity across the chromosome 9q region, potentially conferring fitness advantages (**Supplementary Data 10**; **Supplementary Fig. 16**) [3].

Supplementary Data 10. Somatic *RXRA* mutations detected from targeted DNA sequencing. Variant allele fractions indicate potential copy-neutral loss of heterozygosity in many samples.

Sample	RXRA alteration	Variant allele fraction (%)	Hotspot?
17-045-4th-Tissue-D	Missense p.S330F, p.S400F, p.S427F	93.9	Hotspot
17-045-3rd-Tissue	Missense p.S330F, p.S400F, p.S427F	91.2	Hotspot
BC-007-Tissue	Missense p.S330F, p.S400F, p.S427F	87.6	Hotspot
17-058-Tissue	Missense p.P167L, p.P237L, p.P264L	77.8	
17-045-4th-Tissue-C	Missense p.S330F, p.S400F, p.S427F	72.2	Hotspot
T-024-1st-cfDNA	3'-UTR	65.2	
17-058-1st-cfDNA	Missense p.P167L, p.P237L, p.P264L	64.9	
17-045-2nd-Tissue	Missense p.S330F, p.S400F, p.S427F	54.0	Hotspot
17-045-4th-Tissue-B	Missense p.S330F, p.S400F, p.S427F	50.0	Hotspot
ID8-Tissue	Missense p.S330F, p.S400F, p.S427F	49.1	Hotspot
17-167-2nd-Tissue	Intronic	46.2	
ID2-Tissue	3'-UTR	43.4	
BC-010-Tissue	Intronic	43.1	
BC-005-1st-Tissue	Intronic	43.0	
17-045-1st-Tissue	Missense p.S330F, p.S400F, p.S427F	41.7	Hotspot
17-045-1st-cfDNA	Missense p.S330F, p.S400F, p.S427F	36.4	Hotspot
17-261-1st-Tissue	Missense p.S330Y, p.S400Y, p.S427Y	32.4	Hotspot
ID2-Tissue	3'-UTR	26.7	
17-045-4th-Tissue-A	Missense p.S330F, p.S400F, p.S427F	24.7	Hotspot
ID2-3rd-cfDNA	3'-UTR	23.6	
ID2-2nd-cfDNA	3'-UTR	23.5	

ID2-1st-cfDNA	3'-UTR	23.2	
BC-016-1st-cfDNA	3'-UTR	23.0	
T-022-3rd-cfDNA	Intronic	20.1	
BC-019-Tissue	Synonymous p.S217S, p.S287S, p.S314S	18.6	
BC-019-Tissue	Missense p.S217F, p.S287F, p.S314F	18.2	
ID12-Tissue	3'-UTR	17.8	
17-294-1st-Tissue	3'-UTR	17.4	
T-029-Tissue	Intronic	16.6	
17-225-1st-cfDNA	Intronic	16.5	
ID17-Tissue	3'-UTR	15.2	
17-262-Tissue	Intronic	15.1	
17-294-2nd-Tissue	3'-UTR	11.0	
17-045-2nd-cfDNA	Missense p.S330F, p.S400F, p.S427F	10.6	Hotspot
17-261-2nd-Tissue	Missense p.S330Y, p.S400Y, p.S427Y	10.0	Hotspot
18-080-1st-cfDNA	Intronic	9.8	
BC-010-Tissue	Intronic	9.4	
17-225-2nd-cfDNA	Intronic	6.7	
17-225-3rd-cfDNA	Intronic	6.3	
BC-003-1st-cfDNA	Intronic	5.0	
BC-014-1st-cfDNA	3'-UTR	4.1	
BC-007-1st-cfDNA	Missense p.S330F, p.S400F, p.S427F	3.5	Hotspot
17-167-1st-cfDNA	Intronic	2.3	
17-262-2nd-cfDNA	Intronic	2.1	
17-262-1st-cfDNA	Intronic	2.0	
ID13-2nd-cfDNA	3'-UTR	1.7	
18-262-1st-cfDNA	Intronic	1.7	
ID21-4th-cfDNA	3'-UTR	1.6	
ID21-1st-cfDNA	3'-UTR	1.3	

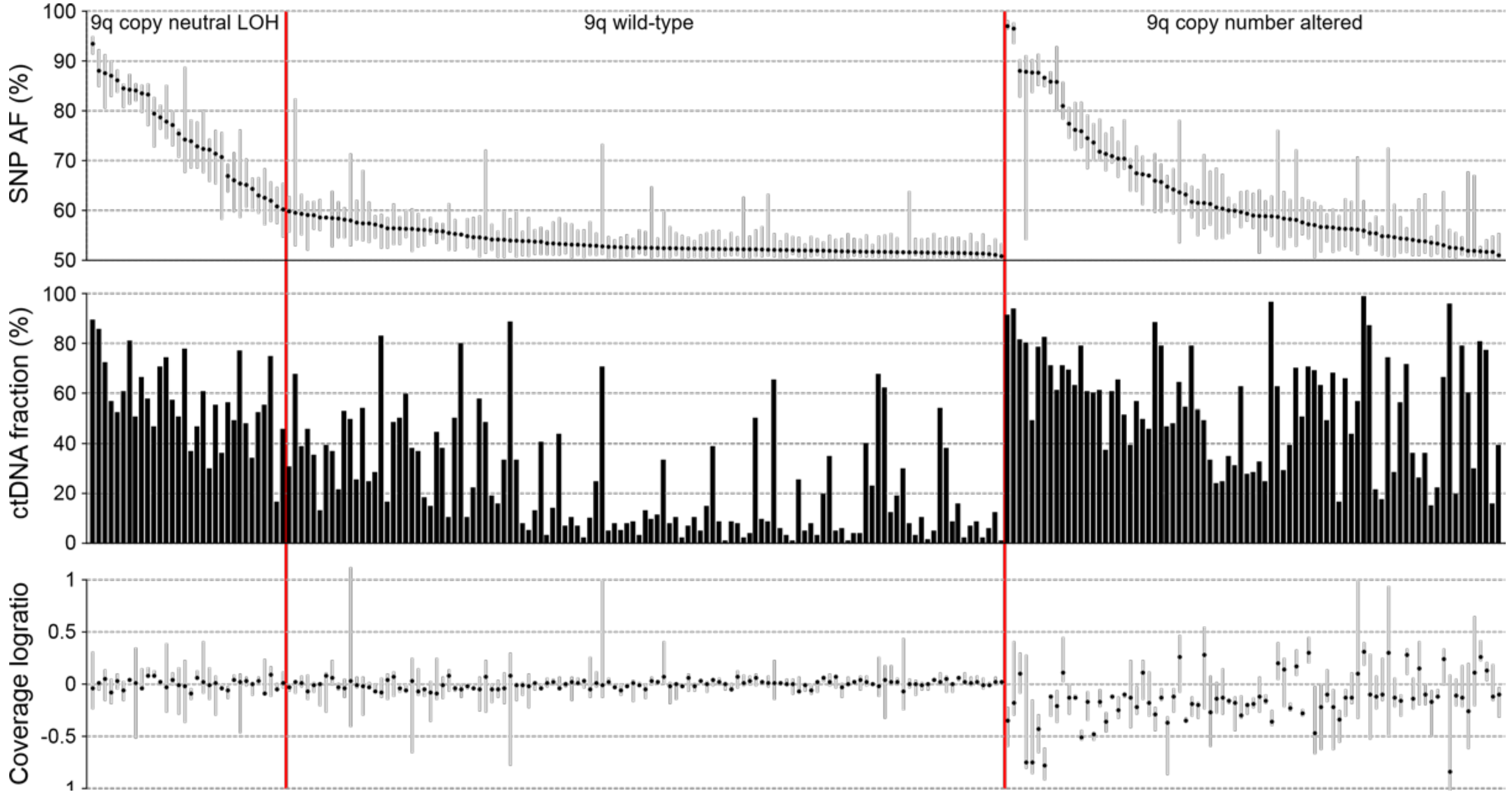

Supplementary Fig. 16. Evidence for frequent loss of heterozygosity (LOH) on chromosome 9q. All circulating tumor DNA (ctDNA) positive samples are included, each represented by a vertical column across the three panels. The median allele fraction (AF) of 9q heterozygous single nucleotide polymorphisms (SNPs) (black dots), and 10% and 90% quantiles (gray lines) are shown in the top panel. The middle panel represents the ctDNA fraction. The bottom panel shows the median coverage logratio of sequenced 9q genes (black dots), and extrema (gray lines). Vertical red lines delineate three groups: samples with 9q copy neutral LOH (median AF of 9q heterozygous SNPs above 60%; coverage logratios between -0.1 and 0.1), samples with wildtype 9q (median AF of 9q heterozygous SNPs between 50 - 60%; coverage logratios between -0.1 and 0.1), and samples with potential 9q copy number alterations (coverage logratios below -0.1 or above 0.1; gain or deletion status not directly inferred due to the complexity of many copy number profiles in mUC). Grouping is uncertain for low ctDNA fraction samples with limited detection sensitivity for coverage logratio changes.

Intra-tumoral heterogeneity

We profiled spatially-distinct regions from cystectomy specimens, and found evidence for spatial intra-tumoral heterogeneity in driver gene alterations in one of three patients (**Supplementary Fig.17**, highlighting the potential for sampling bias when analyzing a single primary tumor foci [4-5].

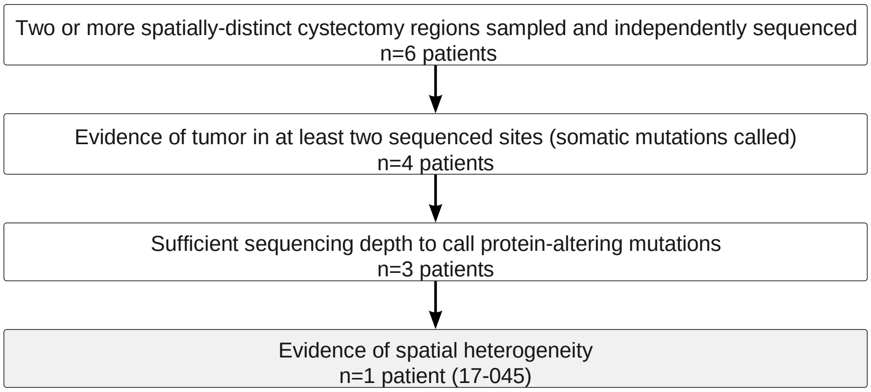

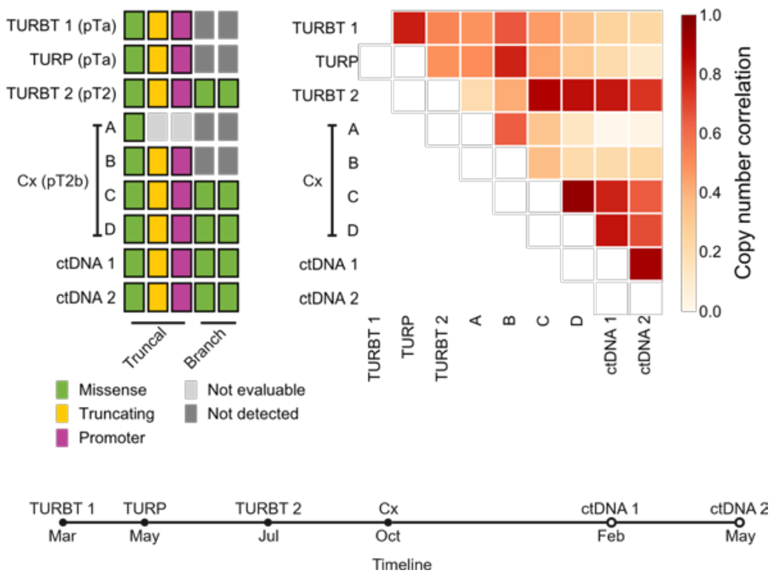

Supplementary Fig. 17. Investigation of intra-tumoral spatial heterogeneity in archival tissue specimens. For six patients, multiple tissue foci received targeted DNA sequencing, and in one patient we identified spatial heterogeneity. Cystectomy (Cx) regions A and B lack mutations in *PIK3CA* and *KMT2D*, consistent with the genomic profiles of prior non-muscle invasive tissue from transurethral resection of the bladder tumor (TURBT) and prostate (TURP). However, a prior muscle-invasive TURBT harbors the branch mutations identified in Cx regions C and D, which are consistent with the clone predominant in later metastatic circulating tumor DNA (ctDNA) samples. These clonal relationships are also evident in the correlation of copy number logratio values across 50 genes. Timeline of sampling is indicated below. Not evaluable indicates insufficient sequencing depth to call a mutation at that site.

Kataegis

In one patient (17-058), tumor mutational burden (TMB) estimates were clearly inflated by local hypermutation, with a kataegic focus within *ARID1A* identified in ctDNA and tissue. Evaluation of the cohort revealed four additional patients with potential kataegic foci (≥6 mutations with an average inter-mutational distance ≤1 Kb; **Supplementary Fig. 18)**.

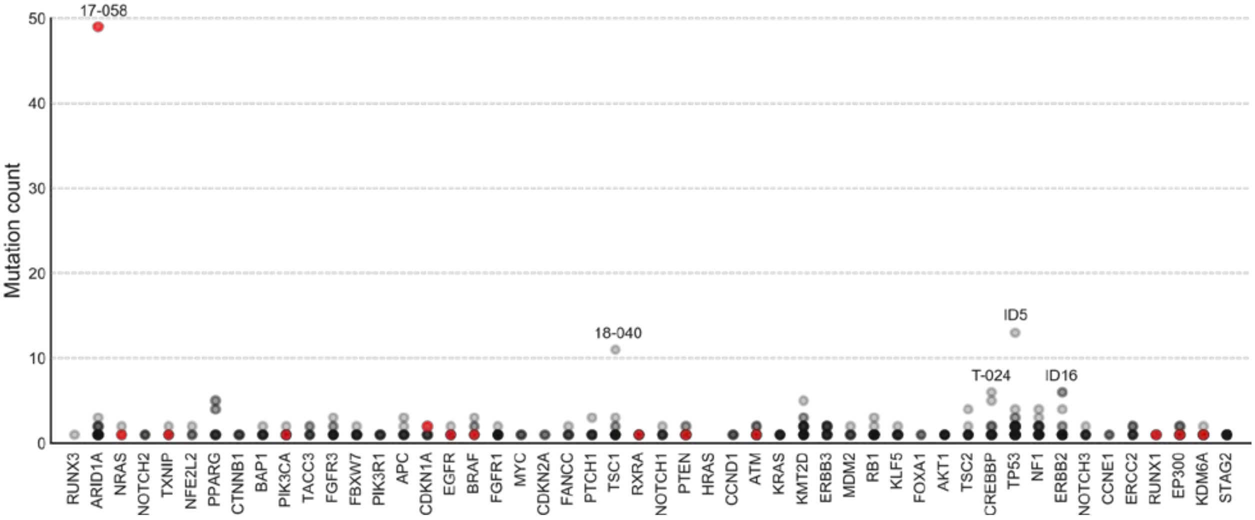

Supplementary Fig. 18. Evidence for kataegis in circulating tumor DNA (ctDNA). Each dot represents the mutation count in that gene for a single patient, as determined from targeted ctDNA sequencing. Red dots correspond to the patient with a clear kataegic focus in *ARID1A*, highlighting normal mutation counts across the remaining genes on the targeted panel. Patients with potential kataegic foci are also annotated.

Supplementary References

1. Canisius, S., Martens, J. W. M. & Wessels, L. F. A. A novel independence test for somatic alterations in cancer shows that biology drives mutual exclusivity but chance explains most co-occurrence. *Genome Biol.* **17**, 261 (2016).

2. Jebar, A. H. *et al.* FGFR3 and Ras gene mutations are mutually exclusive genetic events in urothelial cell carcinoma. *Oncogene* **24**, 5218–5225 (2005).

3. Bielski, C. M. *et al.* Widespread Selection for Oncogenic Mutant Allele Imbalance in Cancer. *Cancer Cell* **34**, 852–862.e4 (2018).

4. Pouessel, D. *et al.* Tumor heterogeneity of fibroblast growth factor receptor 3 (FGFR3) mutations in invasive bladder cancer: implications for perioperative anti-FGFR3 treatment. *Ann. Oncol.* **27**, 1311–1316 (2016).

5. Faltas, B. M. *et al.* Clonal evolution of chemotherapy-resistant urothelial carcinoma. *Nat. Genet.* **48**, 1490–1499 (2016).
