# Supplementary material for: Plasma ctDNA is a tumor tissue surrogate and enables clinical-genomic stratification of metastatic bladder cancer
Source: Nat Commun. 2021 Jan 8;12:184. doi: 10.1038/s41467-020-20493-6 (PMC7794518; doi:10.1038/s41467-020-20493-6)
Supplement: Supplementary file 2 — Description of Additional Supplementary Files [file 41467_2020_20493_MOESM2_ESM.pdf]

Description of Additional Supplementary Files

File Name: Supplementary Data 1-9
Descriptions:

Supplementary Data 1: Tumor samples sequenced from 104 metastatic urothelial carcinoma (mUC) patients and 40 MIBC patients. Information regarding sample type, sequencing performed, sequencing quality metrics, tumor fraction (cancer %), and tumor mutational burden estimates are included.

Supplementary Data 2: Calculation of tumor fraction as a percentage of total DNA. Estimates are based on the highest reliable variant allele frequency (VAF) mutation in each sample unless otherwise stated. Mutations highlighted in red were excluded from the estimation (see Methods).

Supplementary Data 3: Cox proportional-hazards models. Only patients with at least one protein-altering somatic mutation detected are included in analyses incorporating genomic variables.

Supplementary Data 4: Somatic mutations detected from targeted DNA sequencing of the mUC patient cohort. Variant allele fractions are indicated as a percentage, followed by read depth in brackets, with asterisks indicating mutations that met calling criteria in that sample.

Supplementary Data 5: Trinucleotide signature contributions for 35 cell-free DNA samples with whole-exome sequencing.

Supplementary Data 6: Comparison of mutation calls in 46 of the 63 patients with circulating-tumor DNA (ctDNA) and patient-matched tumor tissue. Patients highlighted in grey (n=17) were excluded from analysis due to an insufficient tumor fraction in one/both of the paired samples.

Supplementary Data 7: Somatic protein-altering mutations called across the 46 patient-matched circulating tumor DNA (ctDNA) and tissue pairs. Read support for the 44/265 mutations called exclusively in ctDNA or tissue is compared via Fisher exact test; few discordant mutation calls can be explained by insufficient sequencing depth in the paired sample, as there is a significant difference in read evidence between the two samples.

Supplementary Data 8: Somatic FGFR3 and ERBB2 alterations detected from targeted DNA sequencing. All samples from each patient with an FGFR3 or ERBB2 alteration are shown, regardless of whether the alteration was identified in that sample.

Supplementary Data 9: Somatic rearrangements detected from targeted DNA sequencing.
