# Supplementary material for: Plasma ctDNA is a tumor tissue surrogate and enables clinical-genomic stratification of metastatic bladder cancer
Source: Nat Commun. 2021 Jan 8;12:184. doi: 10.1038/s41467-020-20493-6 (PMC7794518; doi:10.1038/s41467-020-20493-6)
Supplement: Supplementary file 4 — Reporting summary [file 41467_2020_20493_MOESM4_ESM.pdf]

nature research

Corresponding author(s): Alexander W Wyatt and Bernhard J Eigl

Last updated by author(s): Nov 24, 2020

Reporting Summary

Nature Research wishes to improve the reproducibility of the work that we publish. This form provides structure for consistency and transparency in reporting. For further information on Nature Research policies, see our Editorial Policies and the Editorial Policy Checklist.

Statistics

For all statistical analyses, confirm that the following items are present in the figure legend, table legend, main text, or Methods section.

n/a	Confirmed	
☐	☒	The exact sample size (n) for each experimental group/condition, given as a discrete number and unit of measurement
☐	☒	A statement on whether measurements were taken from distinct samples or whether the same sample was measured repeatedly
☐	☒	The statistical test(s) used AND whether they are one- or two-sided<br>*Only common tests should be described solely by name; describe more complex techniques in the Methods section.*
☐	☒	A description of all covariates tested
☐	☐	A description of any assumptions or corrections, such as tests of normality and adjustment for multiple comparisons
☐	☒	A full description of the statistical parameters including central tendency (e.g. means) or other basic estimates (e.g. regression coefficient) AND variation (e.g. standard deviation) or associated estimates of uncertainty (e.g. confidence intervals)
☐	☒	For null hypothesis testing, the test statistic (e.g. F, t, r) with confidence intervals, effect sizes, degrees of freedom and P value noted<br>*Give P values as exact values whenever suitable.*
☒	☐	For Bayesian analysis, information on the choice of priors and Markov chain Monte Carlo settings
☒	☐	For hierarchical and complex designs, identification of the appropriate level for tests and full reporting of outcomes
☐	☒	Estimates of effect sizes (e.g. Cohen's d, Pearson's r), indicating how they were calculated

Our web collection on statistics for biologists contains articles on many of the points above.

Software and code

Policy information about availability of computer code

Data collection	Publicly available TCGA data was downloaded from cBioPortal (see Figure 2 and Methods). For DISCOVER mutual exclusivity analysis (see Supp Results), TCGA BLCA data was downloaded from FireBrowse (version 2016_01_28).
Data analysis	In-house computer code is available at https://github.com/annalam/muc-manuscript-code. Mutational signature analysis of whole-exome sequencing data was performed as described by Rosenthal et al. 2016, using a Python implementation of the deconstructSigs algorithm v1.47 (code available at https://github.com/vanallenlab/deconstruct_sigs_py) and COSMIC mutational signatures v2. Software utilized for analyzing RNA sequencing data include Kallisto 0.45.0, Tximport 1.10.1, and DESeq2 1.22.2 (see Methods). RNA subtype classifications were performed using code provided by Kamoun et al. 2020, available at https://github.com/cit-bioinfo/BLCAsubtyping. As stated in the Supplementary Results, mutual exclusivity testing was performed with DISCOVER v0.9, as described by Canisius et al. 2016 and available at https://github.com/NKI-CCB/DISCOVER#documentation. Survival analysis was performed using Python 3.7.4 with lifelines v0.22.6.

For manuscripts utilizing custom algorithms or software that are central to the research but not yet described in published literature, software must be made available to editors and reviewers. We strongly encourage code deposition in a community repository (e.g. GitHub). See the Nature Research guidelines for submitting code & software for further information.

Data

Policy information about availability of data

All manuscripts must include a data availability statement. This statement should provide the following information, where applicable:

- Accession codes, unique identifiers, or web links for publicly available datasets
- A list of figures that have associated raw data
- A description of any restrictions on data availability

The de-identified sequencing data have been deposited in the European Genome-phenome Archive (EGA) database under the accession code EGAS00001004615

[https://www.ebi.ac.uk/ega/studies/EGAS00001004615]. The TCGA data referenced [https://doi.org/10.1016/j.cell.2017.09.007] are available in public repositories from the cBioPortal [https://www.cbioportal.org] and FireBrowse [http://firebrowse.org] websites. The MSigDB gene sets referenced [https://doi.org/10.1016/j.cels.2015.12.004] are available from the GSEA website [https://www.gsea-msigdb.org/gsea/msigdb]. Source data are provided with this paper; the source data underlying Table 1 and Figures 1-5 are provided as a Source Data file. All the other data supporting the findings of this study are available within the article and its supplementary information/data files, and from the corresponding author upon reasonable request.

Field-specific reporting

Please select the one below that is the best fit for your research. If you are not sure, read the appropriate sections before making your selection.

- [x] Life sciences
- [ ] Behavioural & social sciences
- [ ] Ecological, evolutionary & environmental sciences

For a reference copy of the document with all sections, see nature.com/documents/nr-reporting-summary-flat.pdf

Life sciences study design

All studies must disclose on these points even when the disclosure is negative.

Sample size	The sample size in our retrospective study is comparable or superior to the sample size in other publications investigating mUC clinical outcomes and ctDNA (Chalfin et al. 2019, n=16; Raja et al. 2018, n=29; Grivas et al. 2020, n=124). All blood samples consecutively accrued to our biobank were included, in addition to all equivalent samples available from collaborators, so long as inclusion criteria were met (as specified in Methods) and the patient provided consent. Tissue was retrieved when available within a reasonable timeframe for analysis.
Data exclusions	The exclusion criteria were pre-established. Patients/samples with low tumor fractions (insufficient to detect protein-altering somatic mutations) were excluded from subsequent analyses incorporating genomic variables (e.g. survival regression in Figure 4e, concordance analyses). Patients with incomplete clinical prognostic data were excluded from the multivariate model.
Replication	Due to the limited volumes of plasma/DNA available, and cost considerations, it was not feasible to perform sequencing assays more than once per sample. However, for n=49 samples, targeted and whole exome sequencing were performed using the same DNA library to validate tumor purity and tumor mutational burden estimates.
Randomization	Randomization was not applied in our retrospective sequencing study, as samples from all patients consecutively enrolled to our biobank were included regardless of treatment protocol.
Blinding	Blinding was not applied in our retrospective sequencing study, as samples from all patients consecutively enrolled to our biobank were included regardless of treatment protocol.

Reporting for specific materials, systems and methods

We require information from authors about some types of materials, experimental systems and methods used in many studies. Here, indicate whether each material, system or method listed is relevant to your study. If you are not sure if a list item applies to your research, read the appropriate section before selecting a response.

Materials & experimental systems

n/a	Involved in the study	
x		Antibodies
x		Eukaryotic cell lines
x		Palaeontology and archaeology
x		Animals and other organisms
	x	Human research participants
	x	Clinical data
x		Dual use research of concern

Methods

n/a	Involved in the study	
x		ChIP-seq
x		Flow cytometry
x		MRI-based neuroimaging

Human research participants

Policy information about studies involving human research participants

Population characteristics	The participants were all patients with muscle-invasive or metastatic bladder cancer (urinary bladder and/or upper urinary tract, any histologic variant, at least one distant metastatic lesion). A summary of their characteristics can be found in Table 1.
Recruitment	Patients in our study enrolled in a liquid biobanking initiative on a voluntary basis. Given the ease with which minimally-invasive blood collection can be performed (i.e. inclusive of patients with low volume disease, or poor performance status), our cohort is representative of the general patient population and selection bias is unlikely to impact the results.
Ethics oversight	Study approval was granted by the University of British Columbia Clinical Research Ethics Board, the Ethics Committee of Ghent University Hospital, and the Ethics Commission of the Medical Faculty of the Eberhard-Karls-University Tübingen and University Hospital Tübingen.

Note that full information on the approval of the study protocol must also be provided in the manuscript.

Clinical data

Policy information about clinical studies

All manuscripts should comply with the ICMJEguidelines for publication of clinical research and a completedCONSORT checklist must be included with all submissions.

Clinical trial registration	While our patient inclusion criteria allowed patients to be enrolled on clinical trials, samples for our retrospective study were collected through liquid biobanking initiatives and thus no all-inclusive registration is available.
Study protocol	No trial protocol is available as this is a retrospective clinical sequencing study.
Data collection	Between December 2014 and March 2020, whole blood samples were collected from patients at participating sites in Canada, Belgium, and Germany. Clinical follow-up continued through October 2019 (for mUC patients) or August 2020 (for MIBC patients). Patient-matched archival formalin-fixed paraffin-embedded tissue samples were retrieved when available, dating back to September 2006.
Outcomes	As stated in the Methods: For the MIBC patients, recurrence-free survival was calculated as the time from pre-treatment cfDNA collection to disease recurrence. In the mUC setting, overall survival was defined as either the time from first cfDNA collection to death, or date of metastatic diagnosis to death. Progression-free survival was calculated as the time from treatment initiation to documented clinical or radiological progression, or death. Patients without documented events were censored at the date of last follow-up.
